# Therapeutic applications of the selective high affinity ligand drug SH7139 extend beyond non-Hodgkin’s lymphoma to many other types of solid cancers

**DOI:** 10.18632/oncotarget.27709

**Published:** 2020-09-01

**Authors:** Rod Balhorn, Monique Cosman Balhorn

**Affiliations:** ^1^SHAL Technologies Inc., Livermore, CA 94550, USA

**Keywords:** HLA-DR, selective high affinity ligand, SH7139, SH7129, solid cancers

## Abstract

SH7139, the first of a series of selective high affinity ligand (SHAL) oncology drug candidates designed to target and bind to the HLA-DR proteins overexpressed by B-cell lymphomas, has demonstrated exceptional efficacy in the treatment of Burkitt lymphoma xenografts in mice and a safety profile that may prove to be unprecedented for an oncology drug. The aim of this study was to determine how frequently the HLA-DRs targeted by SH7139 are expressed by different subtypes of non-Hodgkin’s lymphoma and by other solid cancers that have been reported to express HLA-DR. Binding studies conducted with SH7129, a biotinylated analog of SH7139, reveal that more than half of the biopsy sections obtained from patients with different types of non-Hodgkin’s lymphoma express the HLA-DRs targeted by SH7139. Similar analyses of tumor biopsy tissue obtained from patients diagnosed with eighteen other solid cancers show the majority of these tumors also express the HLA-DRs targeted by SH7139. Cervical, ovarian, colorectal and prostate cancers expressed the most HLA-DR. Only a few esophageal and head and neck tumors bound the diagnostic. Within an individual’s tumor, cell to cell differences in HLA-DR target expression varied by only 2 to 3-fold while the expression levels in tumors obtained from different patients varied as much as 10 to 100-fold. The high frequency with which SH7129 was observed to bind to these cancers suggests that many patients diagnosed with B-cell lymphomas, myelomas, and other non-hematological cancers should be considered potential candidates for new therapies such as SH7139 that target HLA-DR-expressing tumors.

## INTRODUCTION

Many of the first targeted therapeutics for treating patients diagnosed with non-Hodgkin’s lymphoma (NHL) were chimeric or humanized mouse monoclonal antibodies that recognized a member of the CD family of cell surface receptors [1–11]. While the clinical use of these antibodies has improved the outcomes of therapy for many NHL patients over the past two decades, advances in our understanding of the molecular basis of this disease and the discovery of new targets and treatment strategies has markedly expanded the range of options now available for lymphoma therapy. Today the pipelines of many pharmaceutical companies contain a wide range of both small molecule and antibody-based drugs that bind to and/or inhibit many cell surface receptors, proteins and enzymes required for tumor growth and proliferation. These pipelines also include a number of immunotherapies that utilize, augment or activate a patient’s own immune system to mount an effective anti-tumor response.

One family of cell surface receptors that play a central role in immunological surveillance and the induction of anti-tumor immunity are the Major Histocompatibility Complex (MHC) proteins. An interesting therapeutic approach that has potential for treating many types of solid cancer has focused on the isolation and identification of tumor peptide antigens presented by the MHC class I [12–17] and MHC class II [18–26] proteins that trigger the activation of lymphocytes and the induction of an effective anti-tumor immune response. The majority of this work is being driven by a growing interest in utilizing these antigens to develop vaccines for melanoma, lung, cervical, and other cancers whose cells express proteins that contain sequence mutations or structural features not found in normal cells.

Another approach that has shown some success in its application to the treatment of B-cell derived malignancies involves the development of antibodies and other drugs that target the MHC class II proteins directly [27–36]. The characterization of one such antibody, Lym-1, led to the discovery that its target, HLA-DR10 and other HLA-DRs containing a common epitope located on its β-subunit [37–39], is expressed by many B-cell derived lymphomas and leukemias. While the clinical trials conducted with Lym-1 have demonstrated the utility of HLA-DR as a target for NHL therapy [29, 30, 32, 33, 40], a number of patients in the trials developed serious adverse effects commonly encountered in antibody therapies. In an effort to create small molecule targeting agents for use in cancer therapy that exhibit the same avidity and selectivity of antibodies but lack human antibody-mouse antibody (HAMA) responses and other antibody induced adverse effects [41–50], a series of selective high affinity ligands (SHALs) were developed to target the HLA-DR10 epitope recognized by Lym-1. This work led to the development of the family of tridentate SHALs, which include SH7139 and SH7129, that are the first of a new class of NHL small molecule drug and diagnostic candidates that target tumor cells expressing HLA-DR [51, 52].

The preclinical testing of SH7139 has not only shown that this drug exhibits remarkable anti-tumor activity in an aggressive Burkitt lymphoma mouse xenograft model [52], but the pre-IND enabling toxicology and safety studies conducted with SH7139 also suggests the drug should have few if any adverse effects and a safety margin that is uncharacteristically large for an oncology drug. During the course of this testing a biotinylated analog of SH7139, SH7129, was developed for use as a potential companion diagnostic to identify NHL patients whose tumors express the HLA-DRs targeted by the drug. Previous analyses of SH7129 binding to a small number of B-cell derived lymphoma cell lines [51] and human [53] and canine [54] tumor biopsy samples have shown the diagnostic selectively binds to lymphoma cells expressing certain HLA-DRs or their canine DLA-DR orthologs. The specific HLA-DRs SH7129 target include HLA-DR7, HLA-DR9, HLA-DR10, HLA-DR11, HLA-DR12, HLA-DR13, HLA-DR15, and HLA-DR16 [53].

While there have been numerous reports of HLA-DR expression by melanomas [21, 55–58], cervical [59–62], ovarian [63–68] prostate [19, 69–73], liver [74–77], kidney [18, 78], bone [79], breast [80–85], esophageal [86–89], head and neck [90–92], bladder [93–96], colorectal [97–101], lung [102–105], pancreatic [106], larynx [107–109], gastric [110–113], glioma [114–116], and thyroid [117–120] cancers, these MHC class II proteins have not been adequately evaluated as potential targets in the treatment of non-hematological cancers. Although it is not entirely clear why tumors derived from tissues of non-lymphoid origin express HLA-DR, the predominant theory for which there is a great deal of experimental support suggests this expression can be initiated in response to tumor infiltration by lymphocytes, macrophages or dendritic cells [84, 96] and the release of cytokines [84, 121] during the inflammation that often accompanies tumor growth and the progression of the disease. Tumor cell lines treated with IFN-γ, a cytokine released by activated T-cells and NK cells [122, 123], have been observed to upregulate their expression of HLA-DR [121, 124–127]. Analyses of tumors obtained from cervical cancer patients have also shown the tumor cells in biopsy tissues with higher IFN-γ concentrations exhibit higher levels of HLA-DR expression, and these patients survive longer and have a lower risk of disease recurrence [128]. Other studies of hepatocellular carcinoma (HCC) patients have demonstrated a positive correlation between lower plasma concentrations of IFN-γ, advanced tumor stage, and higher rates of HCC recurrence [129].

In the study reported here, the biotinylated analog of SH7139 (SH7129) was used as an immunohistochemical-type stain to examine the frequency with which lymphomas and non-hematological solid cancers express the HLA-DRs targeted by SH7139 and to obtain an estimate of their level of expression by quantifying the amount of SH7129 bound to the cells. The results show that each of the NHL subtypes tested, as well as 18 other types of solid cancer, express the HLA-DRs targeted by SH7139. A number of the non-hematological cancers express higher levels of HLA-DR and bind more SH7129 than the B-cell lymphomas and leukemias.

## RESULTS

### SH7129 binding to normal tissue

**Figure 1:**
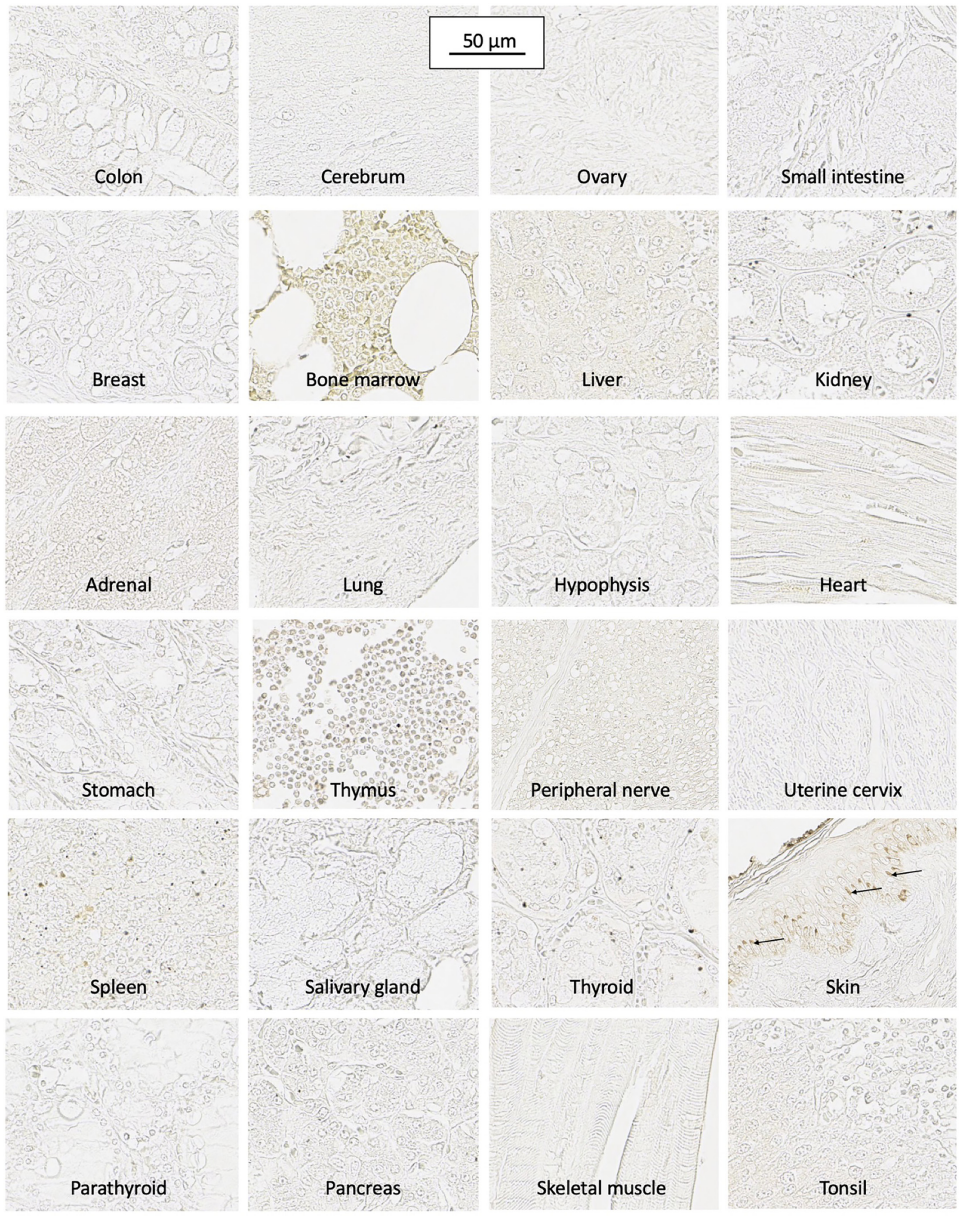
SH7129 binding to normal tissue. Microarrays containing sections from twenty-four normal human tissues (three sections per tissue, each from a different healthy individual) were stained with SH7129 and the bound SHAL was detected using streptavidin conjugated horse-radish peroxidase. Cells binding SH7129 are stained brown. The slides were not counter stained with H&E to enable the detection of very low levels of SH7129 binding. Antigen presenting cells in the bone marrow, thymus, spleen and tonsil bind SH7129 as evidenced by the light staining of cells in those tissues. No binding was observed to breast, cerebrum, colon, hypophysis (pituitary), small intestine, ovary, pancreas, salivary gland, skeletal muscle, thyroid, uterine cervix, peripheral nerve tissue, or skin. The brown coloration in the basal keratinocytes (arrows) in skin is melanin. SH7129 did not bind to lung, esophagus, prostate, cardiac muscle, and parathyroid tissues obtained from two of the three individuals or stomach tissue from one individual. Staining in kidney tissue was limited to the macrophages, dendritic cells and monocytes located between tubules. Liver hepatocytes and zona reticularis cells in the adrenal gland showed very light staining. The images were captured at 40× magnification and the scale bar is the same for all images.

**Figure 2:**
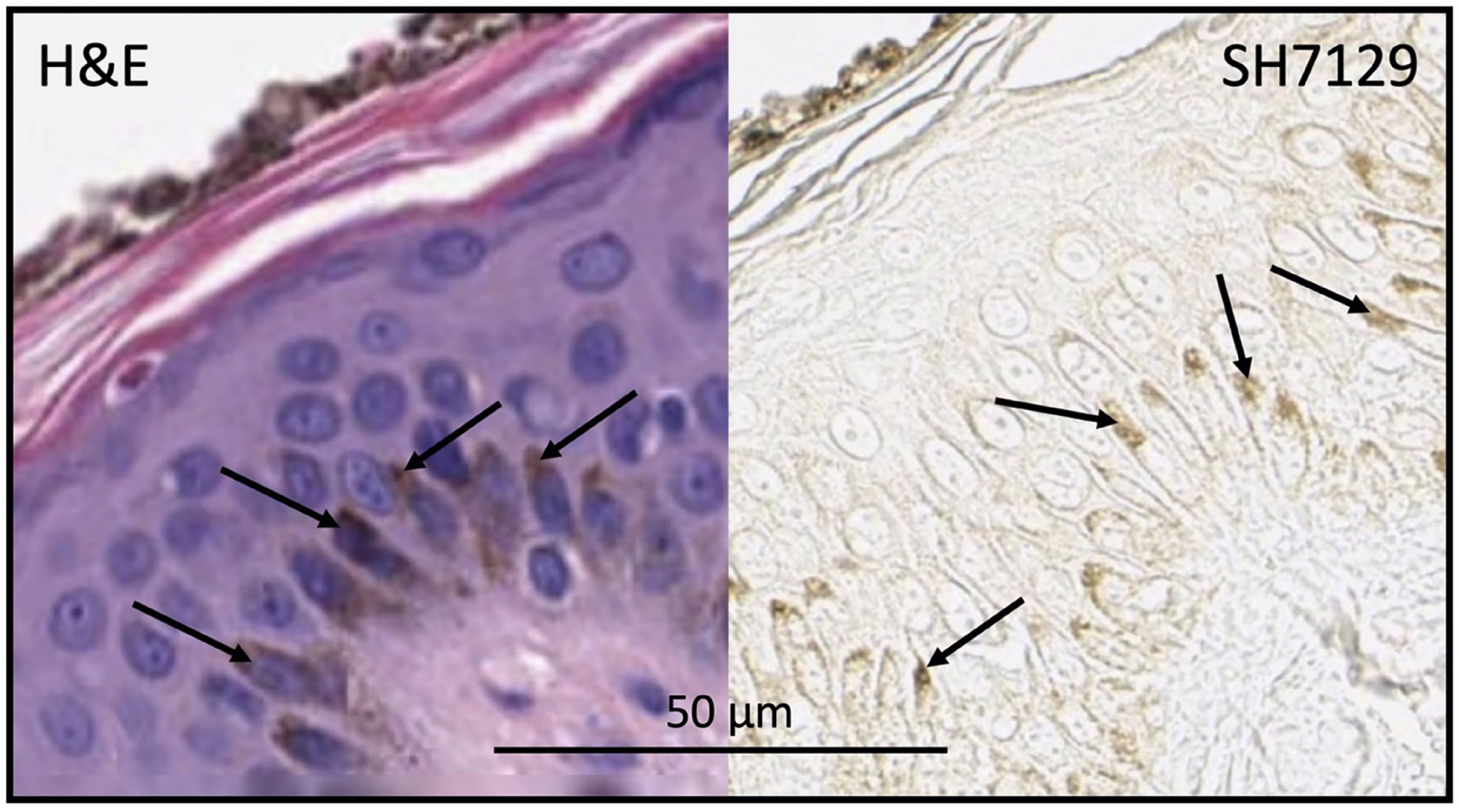
Skin section stained with H&E (without SH7129) or SH7129 (without H&E) showing melanin (arrows) in basal keratinocytes. The apical region of the basal keratinocytes shown in the section stained only with H&E (left panel) has the same brown color as the apical regions of the keratinocytes in the sections stained with SH7129 (right panel). The images were captured at 40× magnification. The scale bar is the same for both images.

SH7129 binding to normal tissue was evaluated using microarrays containing twenty-four different tissues obtained from three healthy individuals. Following the staining of the microarrays with SH7129, the slides were not counter-stained with hematoxylin. This enables the detection of extremely low levels of SH7129 binding that would normally be obscured by the presence of the counter-stain. Cells expressing the target HLA-DRs that bind SH7129 are stained brown by the horse-radish peroxidase’s conversion of the 3,3′-diaminobenzidine (DAB) substrate to a brown insoluble product. As shown in Figure 1, SH7129 binding was observed to tonsil, thymus, spleen, and bone marrow—all tissues that produce or contain large numbers of antigen presenting cells (APCs). No binding was observed to breast, cerebrum, colon, hypophysis (pituitary), small intestine, ovary, pancreas, salivary gland, skeletal muscle, thyroid, uterine cervix, or peripheral nerve tissue. In each of the three skin samples tested the basal keratinocytes appeared to be stained by SH7129, but examination of the control slides (those stained with hematoxylin and eosin without SH7129) revealed this brown coloration is melanin pigment, not bound SH7129 (Figure 2). Some staining was observed in kidney tissue, but the bound SH7129 was limited to the macrophages, dendritic cells and monocytes located between tubules.

**Figure 3:**
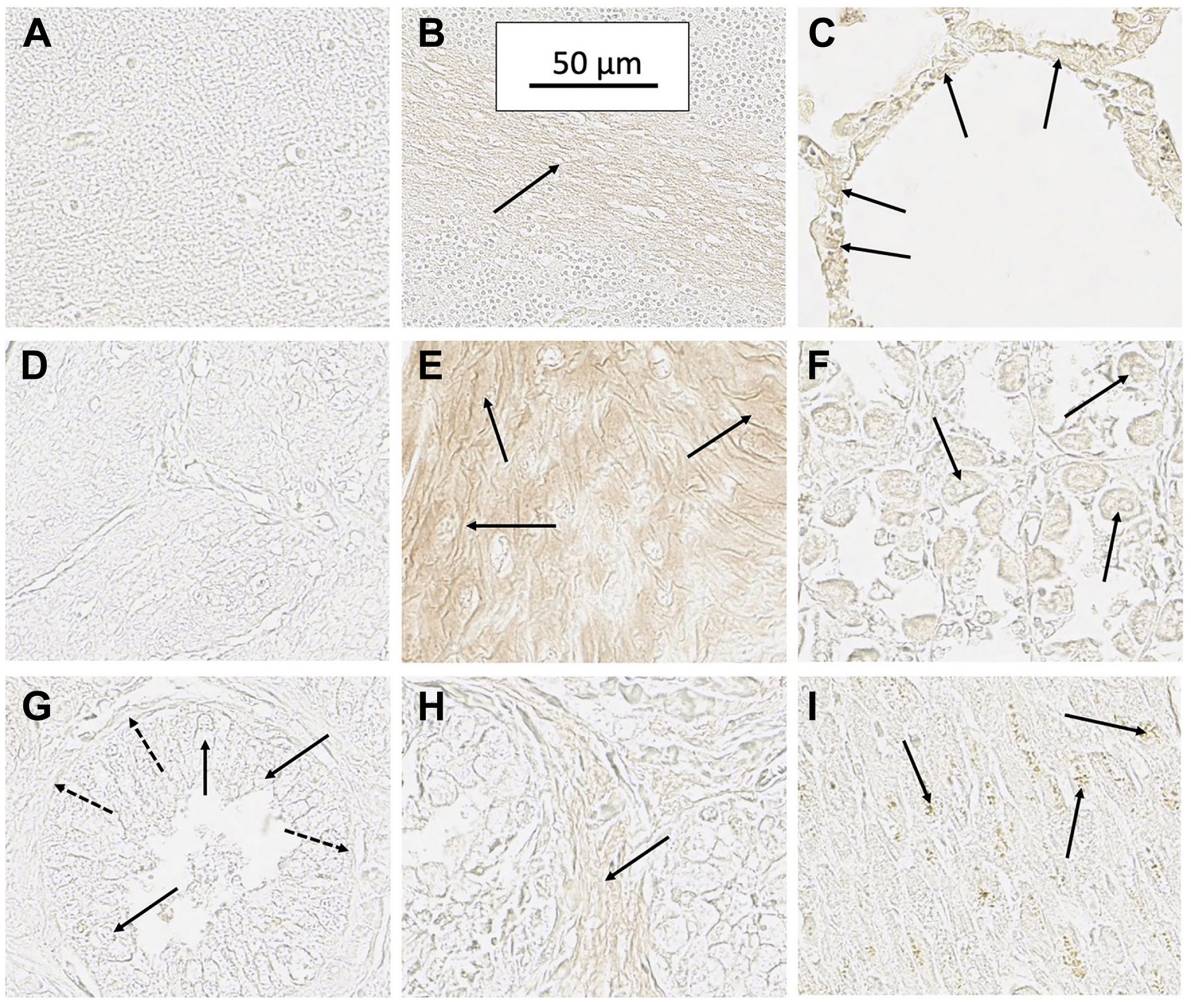
Staining of several normal tissues exhibiting some evidence of SH7129 binding. (**A**) Cerebellum molecular layer (no binding). (**B**) Cerebellum white matter (arrow) showing a low level of SH7129 binding. (**C**) Lung alveolar epithelial cells (arrow) in one section from a normal individual showing SH7129 binding. Lung tissue from two other individuals showed no binding. (**D**) Esophagus tissue (no binding). (**E**) Area in tissue section of esophagus from one individual showing binding to squamous epithelial cells (arrows). Esophageal tissue from other two individuals showed no binding. (**F**) Section of stomach tissue from one individual showing binding to parietal cells (arrows). Stomach tissue from two other individuals showed no binding. (**G**) Section of normal prostate showing no binding to acinar (solid arrows) and basal cells (dashed arrows). (**H**) Section of one normal prostate tissue showing low level binding to stroma (arrow). Prostate tissue from two other individuals showed no binding. (**I**) Section of one normal heart tissue showing very low-level binding to myocytes (arrows). The images were captured at 40× magnification. The scale bar shown in B is the same for all the images.

Cells in the zona reticularis of adrenal tissue were stained very lightly with SH7129, suggesting these cells may also express very low levels of HLA-DR. HLA-DR expression by adrenal cells has been reported previously [130, 131] and it has been suggested that this expression might be induced during the final maturation step for reticularis cells as they become competent to secrete androgens. It has also been suggested the HLA-DR may trigger the induction of apoptosis in these cells *via* MHC class II mediated programmed cell death as part of the normal process of adrenal cell turnover [131]. A very low level of SH7129 binding was also observed in cerebellum white matter (Figure 3). Others who have also reported the binding of anti-HLA-DR antibodies to white matter have suggested this binding may be to resting or non-reactive microglia [132], which are cells of the central nervous system that function as macrophages. HLA-DR expression has been shown to increase in the microglia of individuals diagnosed with the neurogenerative diseases Alzheimer’s, Parkinson’s, and multiple sclerosis as well as in the elderly who do not exhibit dementia [132–138]. Very light staining of parietal cells was also observed in stomach sections (Figure 3) obtained from two individuals, while no staining was observed in the section obtained from the third individual. Parietal cells are epithelial cells that have been reported to express HLA-DR in cases of gastritis [139, 140].

While SH7129 did not bind to lung, esophagus, prostate, cardiac muscle, and parathyroid tissues obtained from two of the three individuals, the tissue from one individual in each case showed very light staining which is just barely detectable in the captured images. Although these tissues do not normally express HLA-DRs [86, 141–144], the low level binding to the tissues from these individuals may reflect undetected tissue inflammation or very early stage disease. In the lung and esophagus tissue section showing staining, SH7129 binding was localized to the epithelial cells in the alveolar ducts of the lung and the squamous epithelium of the esophagus (Figure 3), which are cell types that have been shown to express HLA-DRs during inflammatory lung and esophagus injury or disease [86, 145–152]. The prostate case showed a very light staining of the stroma, while no binding was observed to the acinar or basal cells (Figure 3). In certain cases of chronic immuno-mediated inflammation, such as benign prostatic hyperplasia, prostate stromal cells have been reported to express HLA-DR and function as antigen presenting cells [153, 154]. In the cardiac tissue section from the one individual showing extremely light staining, the binding appeared to be associated with some (not all) of the myocytes (Figure 3)—an observation others have reported to occur in association with transplant tissue [144, 155, 156], myocarditis [157–159] and other types of cardiovascular disease [157, 160, 161]. Thus, the very low level of SH7129 staining of these normal tissues appears consistent with observations in previous studies that have shown HLA-DR expression in healthy lung, esophagus, prostate, cardiac tissue, cerebellum and stomach is confined to non-lymphoid cells that begin functioning as antigen presenting cells in response to injury or disease.

The only other tissue to which SH7129 binding was observed to bind at a very low level was the liver (Figure 1). Hepatocytes in normal liver do not express HLA-DR [162–164], but epithelial cells surrounding portal tracts have occasionally been observed to express HLA-DR in tissue obtained from healthy individuals and much more frequently in cases of disease [165, 166]. Hepatocytes expressing HLA-DR have only been observed in patients with immune mediated liver disorders [164]. Since SH7129 binding was observed in the liver sections from all three individuals and appears to be localized specifically to hepatocytes, it is highly unlikely the tissues were obtained from three individuals that all have a liver disorder. A more likely possibility is that the staining may reflect a low level of SH7129 binding to something other than HLA-DR. Recent studies have shown that SH7139 inhibits OATP1B1 and OATP1B3 [unpublished results], transporters that have only been found in the liver (normal hepatocytes) [167, 168] or certain cancers [168–170]. The abundance of these transporters (3.18 pmoles OATP1B1 and 2.73 pmoles OATP1B3 per 10^6^ hepatocytes [171]) exceed the number of HLA-DR molecules expressed by the Burkitt lymphoma cell line Raji (2.66 pmoles/10^6^ Raji cells [172, 173]) and some of the highest expressing ovarian cancer cells (0.5 pmoles/10^6^ cells [67]) by 2 to 10-fold, respectively. While the binding affinity of SH7129 has not been determined for either transporter, the IC50 for OATP1B1 (0.29 μM) and OATP1B3 (0.15 μM) inhibition by SH7139 indicate SH7129’s affinity for the transporters will be lower than its affinity for HLA-DR (K_D_ = 23 pM [174]). However, SH7129 would be expected to bind to both transporters under the staining conditions used.

### HLA-DR expression by non-Hodgkin’s lymphoma

Using the same SH7129 staining protocol, tumor biopsy sections obtained from patients diagnosed with seven subtypes of NHL were screened for the expression of HLA-DRs targeted by SH7139. Digital images of the tumor cells in each section were obtained from the stained and control (no SH7129 treatment) slides, the images were inverted, and the amount of bound SH7129 was estimated by processing the captured images of each tumor and quantifying the amount of brown oxidized DAB product generated by the horse-radish peroxidase using NIH ImageJ version 1.42 software.

SH7129 staining of biopsy tissues containing cells expressing HLA-DR show binding to HLA-DR proteins located on the surface of the tumor cells, in the cytoplasm, and near the nucleus where the endoplasmic reticulum is located in agreement with previously reported results in cultured Burkitt lymphoma (Raji) cells [51, 175]. Connective tissue is not stained. As shown in Table 1, a significant number of the tested tumors in each of the types of NHL examined were found to bind SH7129. Tumor biopsies obtained from all twenty-four of the anaplastic large cell lymphoma (ALCL) cases examined expressed the targeted HLA-DR and bound SH7129. Nearly every MALT lymphoma (75 of the 80 cases) biopsy sample examined also bound the diagnostic. At the other end of the spectrum, only 28% of the mantle cell and 34% of the follicular lymphomas were observed to express the target and bind SH7129.

**Table 1:** Solid cancers tested for SH7129 binding as an indicator of their expression of the HLA-DRs targeted by SH7139

**Cancer Type**	**Number of Cases**	**Percent Expressing Target**
*Non-Hodgkin’s Lymphoma*		
DLBC Lymphoma	75	57.3
Follicular Lymphoma	118	33.9
Anaplastic LC Lymphoma	24	100
MALT Lymphoma	80	93.8
Mantle Cell Lymphoma	71	28.2
Burkitt Lymphoma	25	32.0
Small Lymphocytic Lymphoma	78	60.2
*Other Cancers*		
Ovarian	64	100
Lung	85	98.8
Cervical	66	98.4
Pancreatic	89	65.1
Gastric	90	91.1
Esophageal	99	2
Breast, Medullary Carcinoma	77	66.2
Breast, Invasive Ductal	90	4.4
Kidney	69	79.7
Prostate	72	95.8
Thyroid	80	65
Liver	75	89.3
Colorectal	92	94.5
Bone	78	69.2
Bladder	60	61.7
Plasma Cell Myeloma	14	92.9
Larynx	70	48.6
Melanoma	122	46.7
Head and Neck	40	5

**Figure 4:**
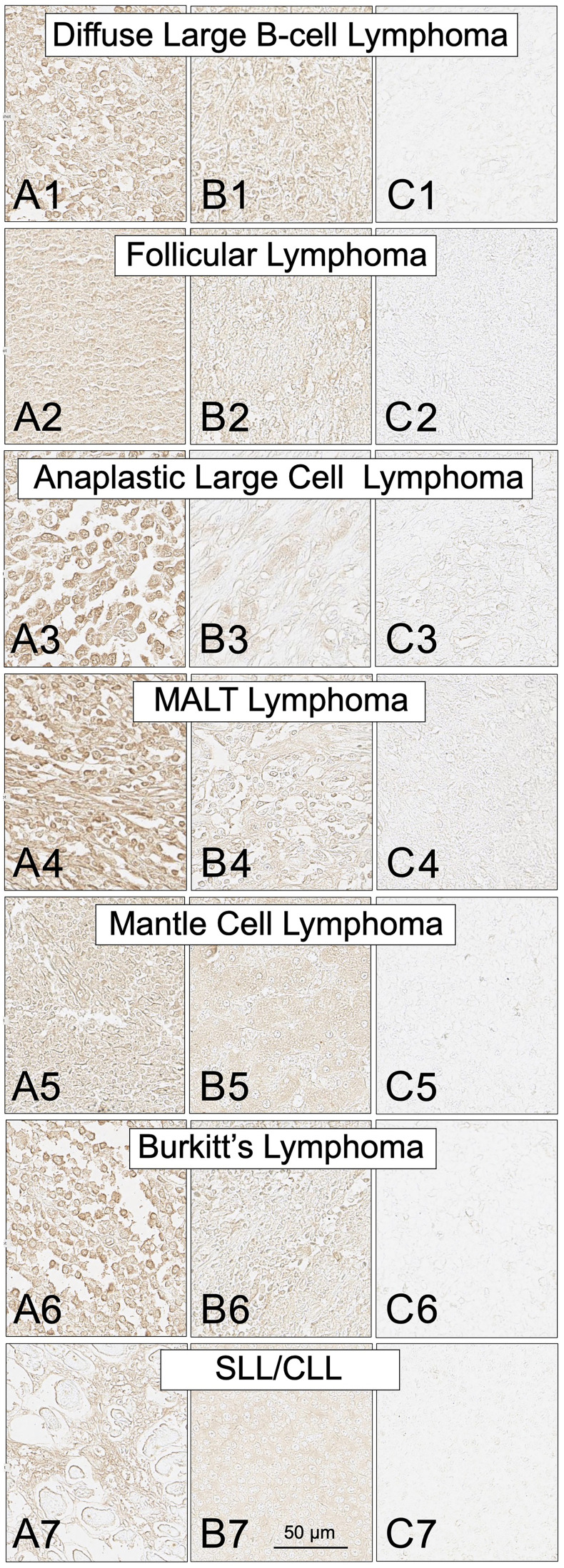
SH7129 staining of tumor biopsy sections from seven subtypes of non-Hodgkin’s lymphoma. Sections from three different tumors are shown for each subtype: high level of SH7129 binding (A), moderate level of SH7129 binding (B), and no SH7129 binding (C). Diffuse large B-cell lymphoma: (**A1**) Tissue sample ODCTLYMLY02E3. (**B1**) Tissue sample LM482C8. (**C1**) Tissue sample LM482C5. Follicular Lymphoma: (**A2**) Tissue sample T203B6. (**B2**) Tissue sample 6050459. (**C2**) Tissue sample 1051864. Anaplastic large cell lymphoma: (**A3**) Tissue sample LM242D5. (**B3**) Tissue sample LM242D4. (**C3**) Tissue sample LM242B1. Mucosa-associated lymphoid tissue lymphoma: (**A4**) Tissue sample LY804E7. (**B4**) Tissue sample LY804A6. (**C4**) Tissue sample LY804G5. Mantle cell lymphoma: (**A5**) Tissue sample M685565. (**B5**) Tissue sample 15726/09. (**C5**) Tissue sample F721798. Burkitt lymphoma: (**A6**) Tissue sample LM482E3. (**B6**) Tissue sample BL1A8. (**C6**) Tissue sample BL1A1. Small lymphocytic lymphoma/chronic lymphocytic leukemia: (**A7**) Tissue sample F683134. (**B7**) Tissue sample 9543432. (**C7**) Tissue sample M672708. The images were captured at 40× magnification. The scale bar is the same for all images.

**Figure 5:**
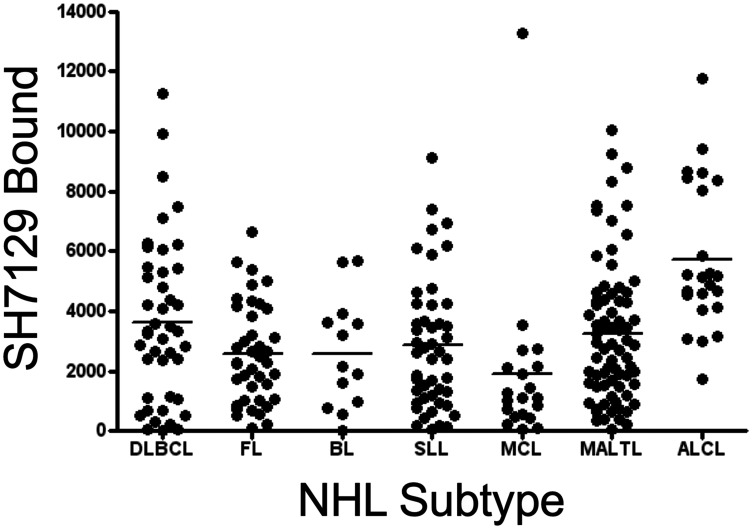
Tumor to tumor variability in SH7129 binding to seven types of NHL. Tumor microarrays containing biopsy tissue sections from tumors of patients diagnosed with diffuse large B-cell lymphoma (DLBCL), follicular lymphoma (FL), Burkitt lymphoma (BL), small lymphocytic lymphoma (SLL), mantle cell lymphoma (MCL), mucosa-associated lymphoid tissue lymphoma (MALTL), and anaplastic large cell lymphoma (ALCL) were stained with SH7129 and the biotin in the bound SHAL was detected using SAHRP and the substrate DAB. The amount of bound SH7129 was determined by densitometric analysis of each tumor section. Not all biopsies contained tumor cells expressing the HLA-DR target to which SH7129 binds. Only those tumors exhibiting SH7129 binding are included in the plot. The value plotted is the amount of bound SH7129 (integrated staining density) per 384-pixel area of tumor tissue. Each point corresponds to a tumor from a different patient. A significant number of tumors representing each subtype of NHL were observed to bind SH7129. The range in SH7129 binding to the tumors within each NHL subtype varied by as much as 100-fold.

Within a typical lymphoma biopsy section, the cell to cell variation in SH7129 binding, as measured by image analysis of the horse-radish peroxidase generated oxidation product of DAB deposited in individual tumor cells, was less than 3-fold (Table 2). The level of HLA-DR expression and SH7129 binding by the cells in different patient’s tumors within the same type of NHL, however, differed by as much as 10 to 100-fold (Figures 4 and 5). Statistical analyses of SH7129 bound by the seven types of NHL indicate these NHL tumors fall into three groups. Biopsy tissues obtained from patients diagnosed with anaplastic large cell lymphoma (ALCL) exhibited the highest level of SH7129 binding. Diffuse large B-cell lymphomas (DLBCL), small lymphocytic lymphomas (SLL), and mucosa-associated lymphoid tissue (MALT) lymphomas bound intermediate amounts of SH7129. Follicular lymphomas (FL), Burkitt’s lymphomas (BL) and mantle cell lymphomas (MCL) bound the least SH7129 of the types of NHL analyzed.

**Table 2:** Cell to cell variation in SH7129 binding in a typical NHL tumor

Lymphoma Type	Biopsy Sample ID	Level of SH7129 Binding	Cell Range SH7129 Bound
Diffuse Large B-Cell	LM482E3	High	2.98
Follicular	T203B6	Moderate	1.69
Burkitt’s	LM482D8	Moderate	2.42
MALT	LY804E7	High	1.94
Anaplastic Large Cell	LM242D5	High	2.75
Mantle Cell	MC2B9	Moderate	1.78

Bound SH7129 was quantified by image analysis for 50 cells representing the full range of binding within a biopsy sample obtained from patients diagnosed with each of six subtypes of NHL. The Level of SH7129 Binding is a subjective descriptor of the overall intensity of SH7129 staining of the tumor tissue relative to other tumors in that subtype. Cell Range in SH7129 Bound is the amount of SH7129 bound by the most intense staining tumor cell divided by the amount of SH7129 bound by the least intense staining tumor cell in the tumor section.

### Identification of other solid cancers that also express HLA-DRs targeted by SH7139

**Figure 6:**
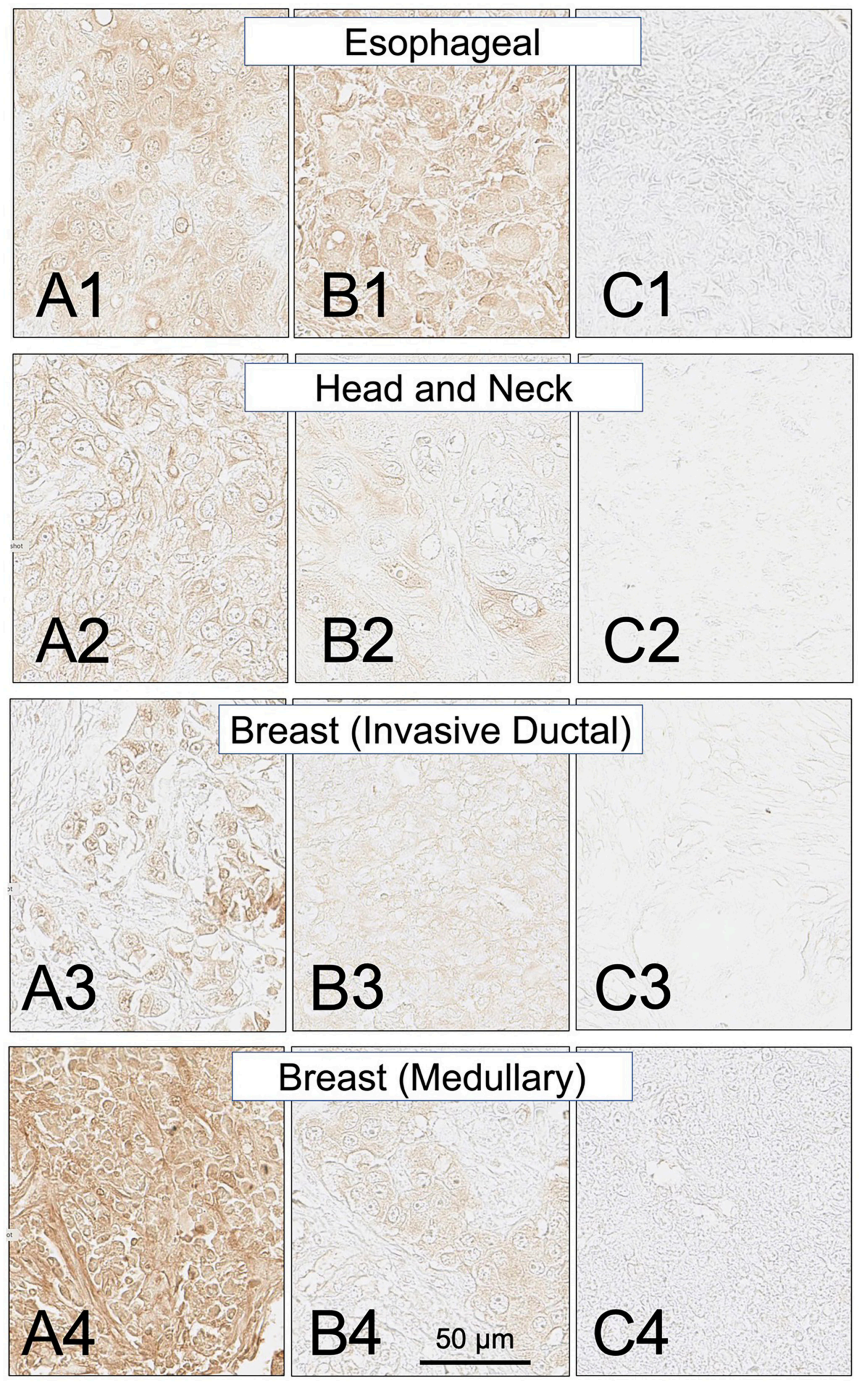
SH7129 stained sections of representative esophageal, head and neck, and breast (invasive ductal and medullary carcinoma) cancers expressing HLA-DRs targeted by SH7139. Sections from three different tumors are shown for each cancer: high level of SH7129 binding (A), moderate level of SH7129 binding (B), and no SH7129 binding (C). Esophageal cancer: (**A1**) Tissue sample ESC1021H1, adenocarcinoma. (**B1**) Tissue sample EC1021A12, squamous cell carcinoma. (**C1**) Tissue sample ESC1021B2, squamous cell carcinoma. Head and Neck: (**A2**) Tissue sample HN483C3, squamous cell carcinoma. (**B2**) Tissue sample HN483A4, squamous cell carcinoma. (**C2**) Tissue sample HN483E5, squamous cell carcinoma. Invasive Ductal breast cancer: (**A3**) Tissue sample BC08118A9. (**B3**) Tissue sample BC08118A5. (**C3**) Tissue sample BC08118H3. Medullary breast cancer: (**A4**) Tissue sample BR807A1. (**B4**) Tissue sample BR807A2. (**C4**) Tissue sample BR807B8. The images were captured at 40× magnification. The scale bar is the same for all images.

**Figure 7:**
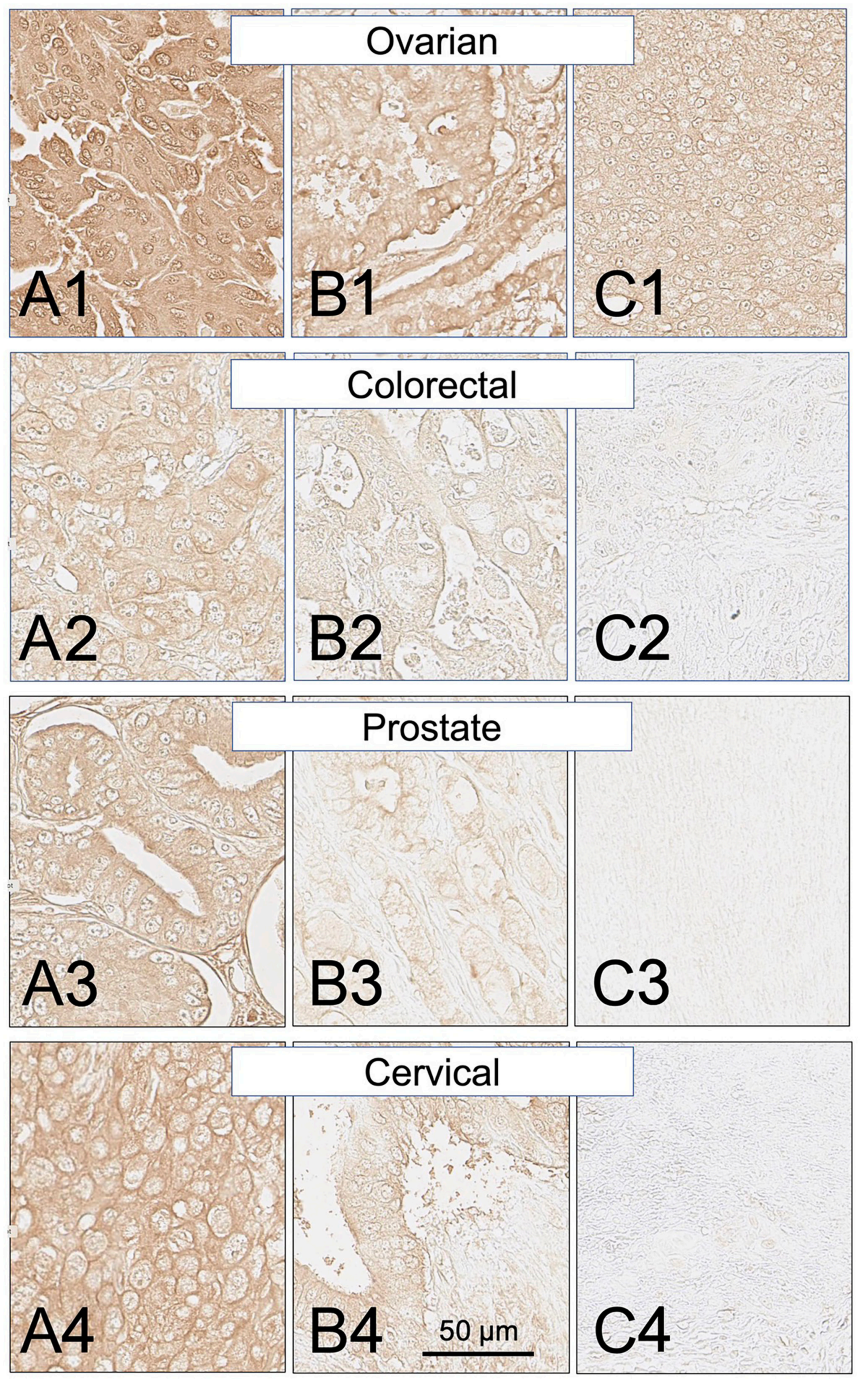
SH7129 stained sections of representative ovarian, colorectal, prostate, and cervical cancers expressing HLA-DRs targeted by SH7139. Three examples are shown for each cancer: high level of SH7129 binding (A), moderate level of SH7129 binding (B), and no SH7129 binding (C). Since all of the tested ovarian cancer biopsy sections bound SH7129, the ovarian cancer example shown in (C) is a third tumor showing SH7129 binding. Ovarian cancer: (**A1**) Tissue sample OVC1501E4, endometrioid adenocarcinoma. (**B1**) Tissue sample OVC1501D1, mucinous cystadenocarcinoma. (**C1**) Tissue sample OVC1501G4, serous cystadenocarcinoma. Colorectal cancer: (**A2**) Tissue sample ODCTDGCOL04D1, adenocarcinoma. (**B2**) Tissue sample ODCTDGCOL04B3, adenocarcinoma. (**C2**) Tissue sample ODCTDGCOL04F8, adenocarcinoma. Prostate cancer: (**A3**) Tissue sample PR803CA10, adenocarcinoma. (**B3**) Tissue sample PR803CE1. (**C3**) Tissue sample PR803CH2. Cervical cancer: (**A4**) Tissue sample CXC1501C6, squamous cell carcinoma. (**B4**) Tissue sample CXC1501G3, adenocarcinoma. (**C4**) Tissue sample CXC1501F6, squamous cell carcinoma. The images were captured at 40× magnification. The scale bar is the same for all images.

**Figure 8:**
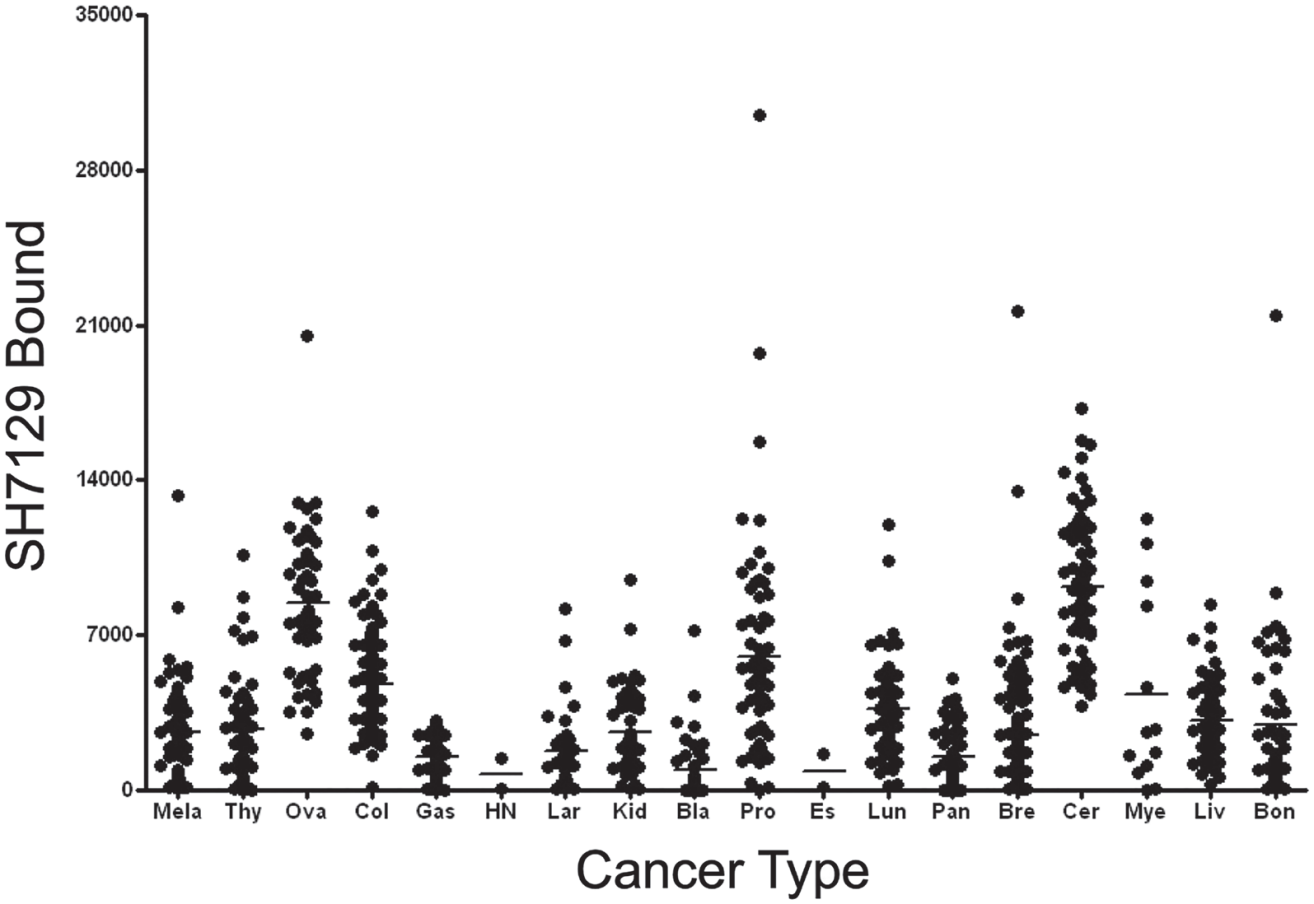
Tumor to tumor variability in SH7129 binding to other cancers. Tumor microarrays containing biopsy sections from tumors of patients diagnosed with one of eighteen different types of solid cancers were stained with SH7129 and the biotin in the bound SHAL was detected using SAHRP and the substrate DAB. The amount of bound SH7129 was determined by densitometric analysis of each tumor section. Not all biopsies contained tumor cells expressing the HLA-DR target to which SH7129 binds. Only those tumors exhibiting SH7129 binding are included in the plot. The value plotted is the amount of bound SH7129 (integrated stain density) per 384-pixel area of tumor tissue. Each point corresponds to a tumor from a different patient. A significant number of tumors representing 16 of the 18 cancers analyzed were observed to bind SH7129. Only two of the head and neck and two esophageal cancers bound SH7129. While the range in SH7129 binding to different cervical, ovarian, breast or myeloma tumors expressing the HLA-DR target is small (5 to 16-fold), SH7129 binding to individual tumors in many of the other non-hematological cancers vary by more than 100-fold. The solid cancers tested include: melanoma (Mela), thyroid (Thy), ovarian (Ova), colorectal (Col), gastric (Gas), head and neck (HN), laryngeal (Lar), kidney (Kid), bladder (Bla), prostate (Pro), esophageal (Es), lung (Lun), pancreatic (Pan), breast (Bre), cervical (Cer), plasma cell myeloma (Mye), liver (Liv), and bone (Bon).

**Figure 9:**
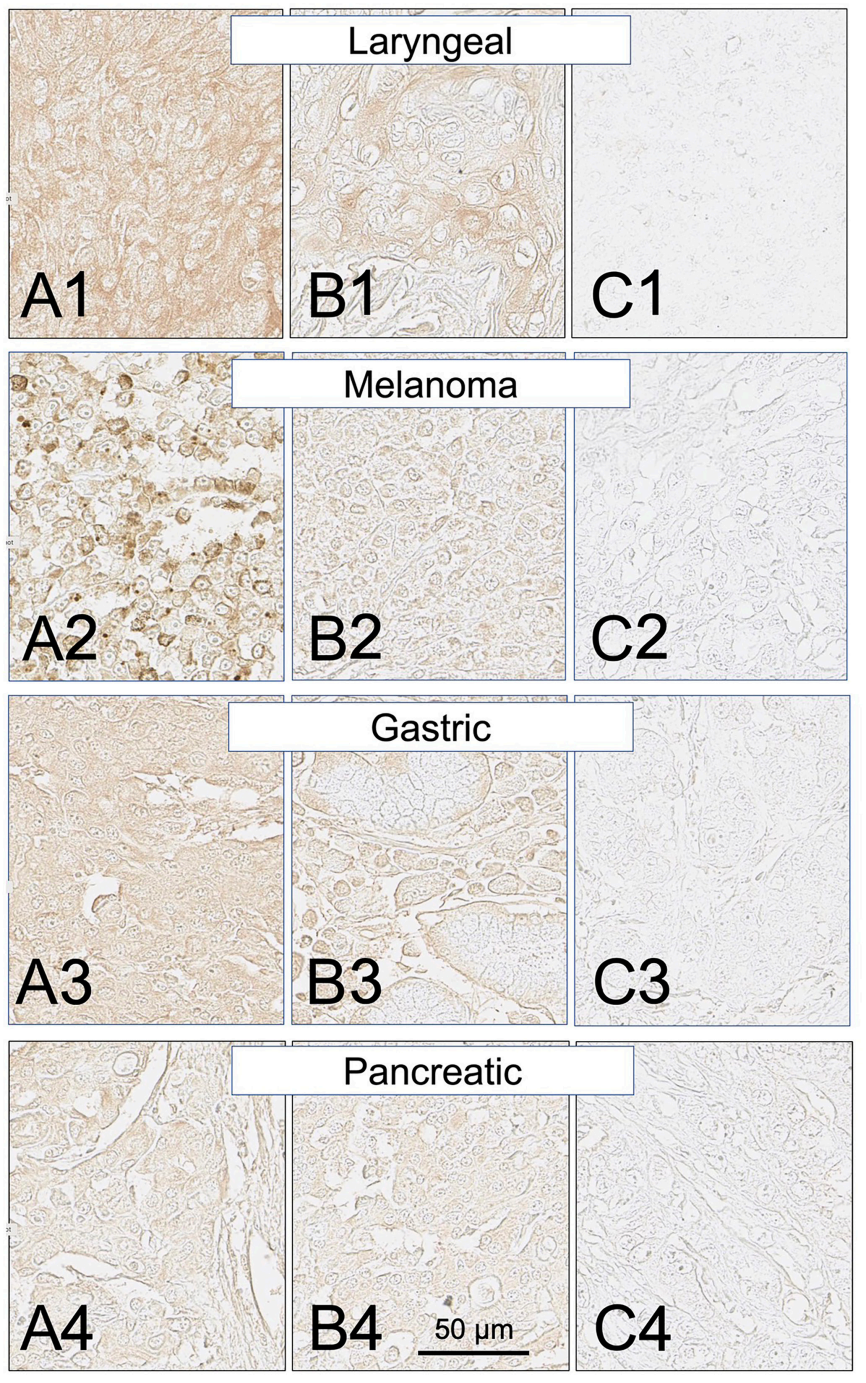
SH7129 stained sections of representative laryngeal, melanoma, gastric, and pancreatic cancers expressing HLA-DRs targeted by SH7139. Sections from three different tumors are shown for each cancer: high level of SH7129 binding (A), moderate level of SH7129 binding (B), and no SH7129 binding (C). Laryngeal cancer: (**A1**) Tissue sample LP801A7, squamous cell carcinoma. (**B1**) Tissue sample LP801C6, basaloid squamous cell carcinoma. (**C1**) Tissue sample LP801G1, squamous cell carcinoma. Melanoma: (**A2**) Tissue sample Me1004EA10. (**B2**) Tissue sample Me1004EG6. (**C2**) Tissue sample Me1004 EB1. Gastric cancer: (**A3**) Tissue sample ODCTDGSTM01E4, adenocarcinoma. (**B3**) Tissue sample ODCTDGSTM01J4, ring cell carcinoma. (**C3**) Tissue sample ODCTDGSTM01I9, adenocarcinoma. Pancreatic cancer: (**A4**) Tissue sample PA961CF1, duct adenocarcinoma. (**B4**) Tissue sample PA961CH2, carcinoid. (**C4**) Tissue sample PA961CF10, adenocarcinoma. The images were captured at 40× magnification. The scale bar is the same for all images.

**Figure 10:**
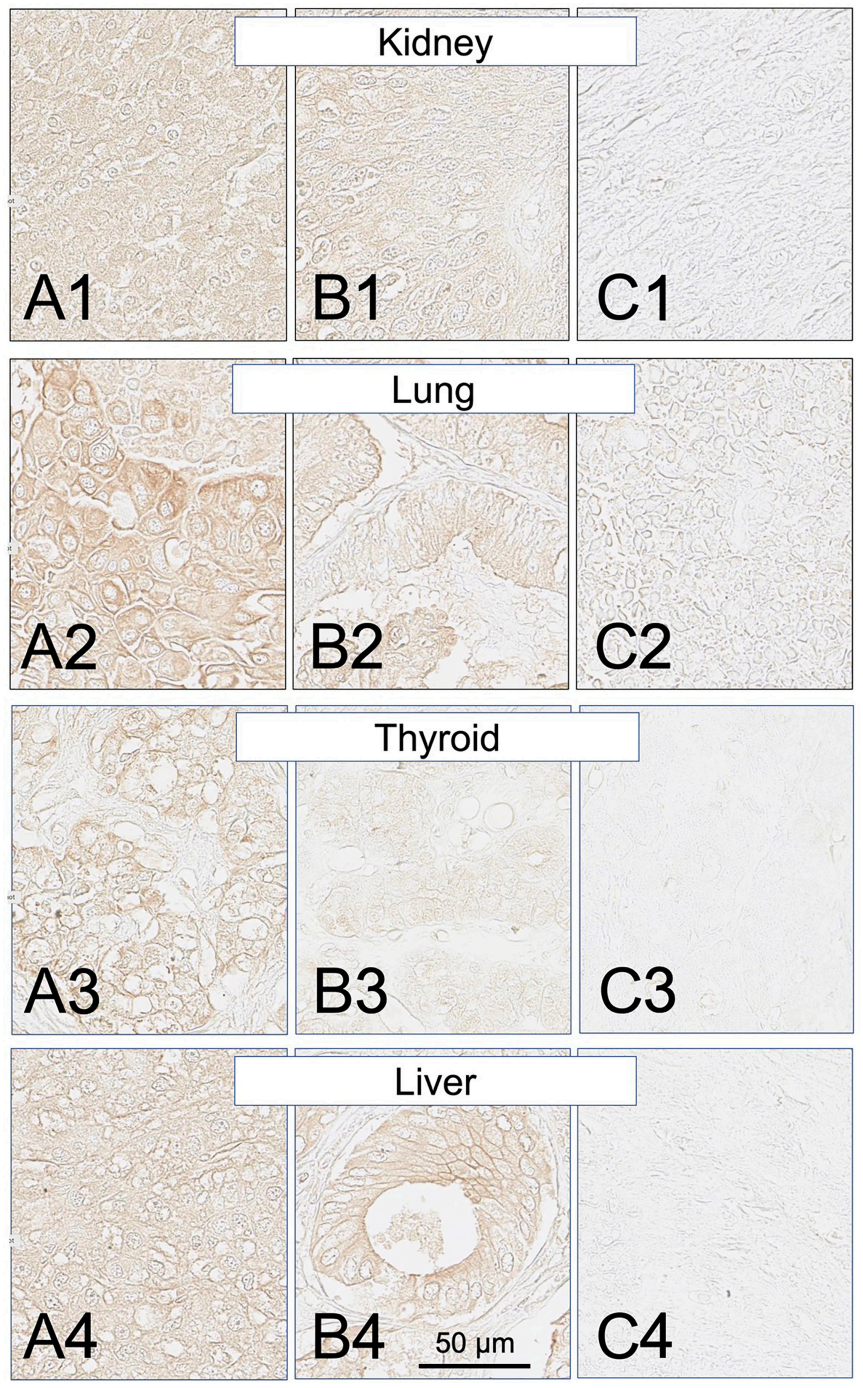
SH7129 stained sections of representative kidney, lung, thyroid and liver cancers expressing HLA-DRs targeted by SH7139. Sections from three different tumors are shown for each cancer: high level of SH7129 binding (A), moderate level of SH7129 binding (B), and no SH7129 binding (C). Kidney cancer: (**A1**) Tissue sample BC07014B1, clear cell carcinoma. (**B1**) Tissue sample BC07014AH2, transitional cell carcinoma. (**C1**) Tissue sample BC07014AB7, clear cell carcinoma. Lung cancer: (**A2**) Tissue sample LUC1021E1, squamous cell carcinoma. (**B2**) Tissue sample LUC1021C1, adenocarcinoma. (**C2**) Tissue sample LUC1021E3, squamous cell carcinoma. Thyroid cancer: (**A3**) Tissue sample TH802C1, papillary carcinoma. (**B3**) Tissue sample TH802E6, follicular adenoma. (**C3**) Tissue sample TH802G7, embryonic adenoma. Liver cancer: (**A4**) Tissue sample LVC1501A9, hepatocellular carcinoma. (**B4**) Tissue sample LVC1501A8, bile duct carcinoma. (**C4**) Tissue sample LVC1501F7, hepatocellular carcinoma. The images were captured at 40× magnification. The scale bar is the same for all images.

**Figure 11:**
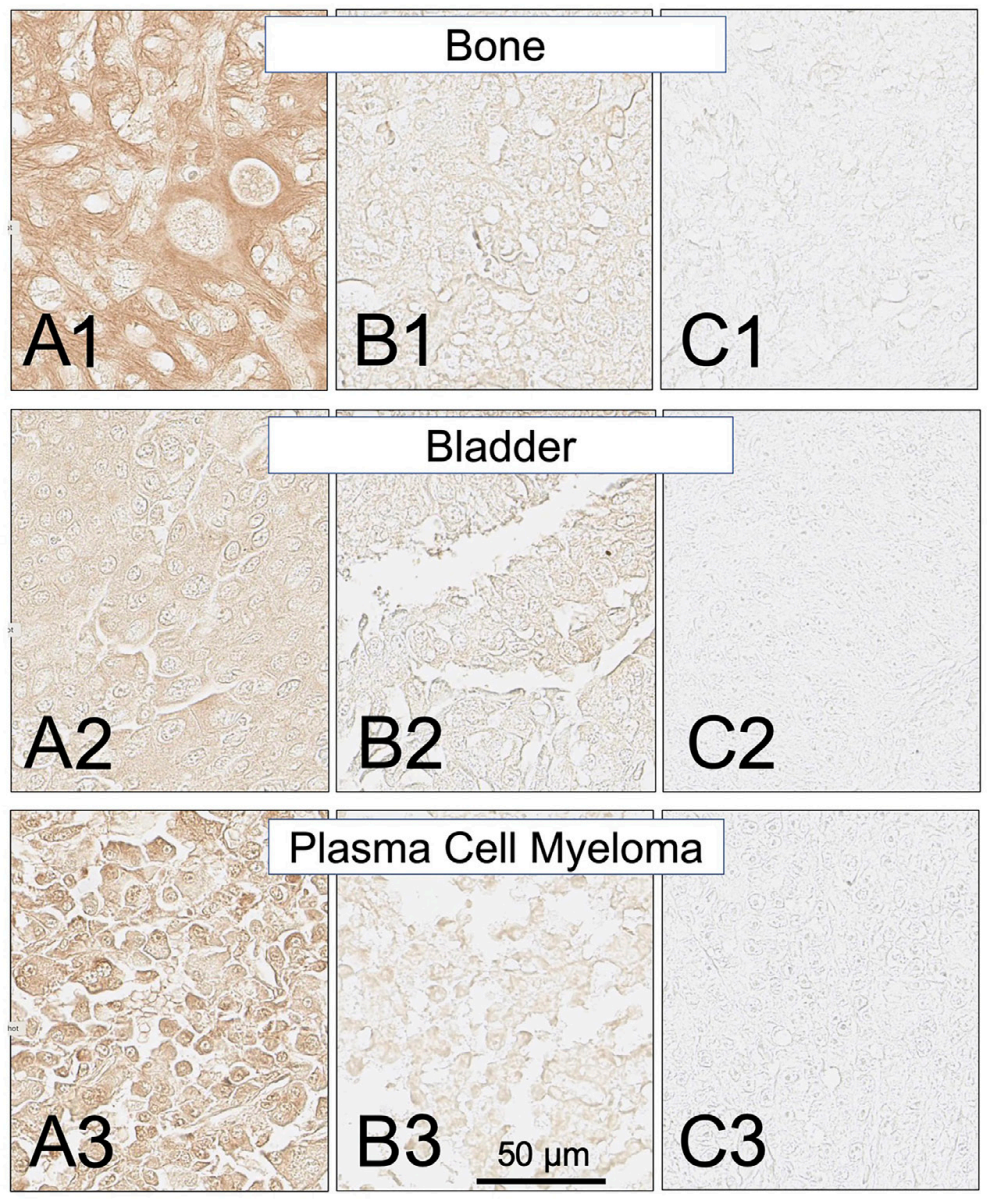
SH7129 stained sections of representative bone, bladder and plasma cell myeloma cancers expressing HLA-DRs targeted by SH7139. Sections from three different tumors are shown for each cancer: high level of SH7129 binding (A), moderate level of SH7129 binding (B), and no SH7129 binding (C). Bone cancer: (**A1**) Tissue sample OS802F5, chondrosarcoma. (**B1**) Tissue sample OS802B8, osteosarcoma. (**C1**) Tissue sample OS802C6, osteosarcoma. Bladder cancer: (**A2**) Tissue sample BC12011BD3, urothelial carcinoma. (**B2**) Tissue sample BC12011BE4, urothelial carcinoma (**C2**) Tissue sample BC12011E6, urothelial carcinoma. Plasma cell myeloma: (**A3**) Tissue sample T291B3. (**B3**) Tissue sample LM482A6. (**C3**) Tissue sample LM482A1. The images were captured at 40× magnification. The scale bar is the same for all images.

Biopsy sections from eighteen additional solid cancers that have been reported by others to express MHC class II proteins were also examined for expression of the HLA-DRs targeted by SH7139 using the same staining protocol. While many (33–100%) of the tumors analyzed in sixteen of these cancers were found to bind SH7129 (Table 1), only two of the ninety-nine esophageal (2%) and two of the forty head and neck tumors (5%) (Figure 6) showed detectable SH7129 binding. The highest percentage of cases showing binding was observed for ovarian, lung, cervical, gastric, prostate, myeloma and colorectal cancers. Ovarian, colorectal, prostate and cervical cancers (Figure 7) exhibited the highest levels of SH7129 binding of all the solid tumors examined (Figure 8). In marked contrast to the other types of cancer, which had cases representing the full spectrum (low to high) of HLA-DR expression and SH7129 binding (Figures 9–11), all of the ovarian and all but one of the cervical cancer biopsy sections examined bound moderate to high levels of SH7129. The amount of SH7129 bound by the two esophageal and two head and neck tumors were amongst the lowest of all the cancers tested.

Similar to the results obtained in the analysis of the different subtypes of NHL, a broad range in the level of SH7129 binding/HLA-DR target expression was observed between individual tumors for many of these solid cancers. The gastric and pancreatic cancer cases (Figures 8 and 9) showed the least variability in SH7129 binding. Within the ovarian, prostate, melanoma, breast and bone cancers the tumors from a small number of outlier cases expressed extremely high levels of the HLA-DR target (Figure 8). The amount of SH7129 bound by these outliers was nearly twice the amount bound by the lymphoma outliers (Figure 5).

### Variation in SH7129 binding by tumor type or grade within the non-hematological cancers

**Figure 12:**
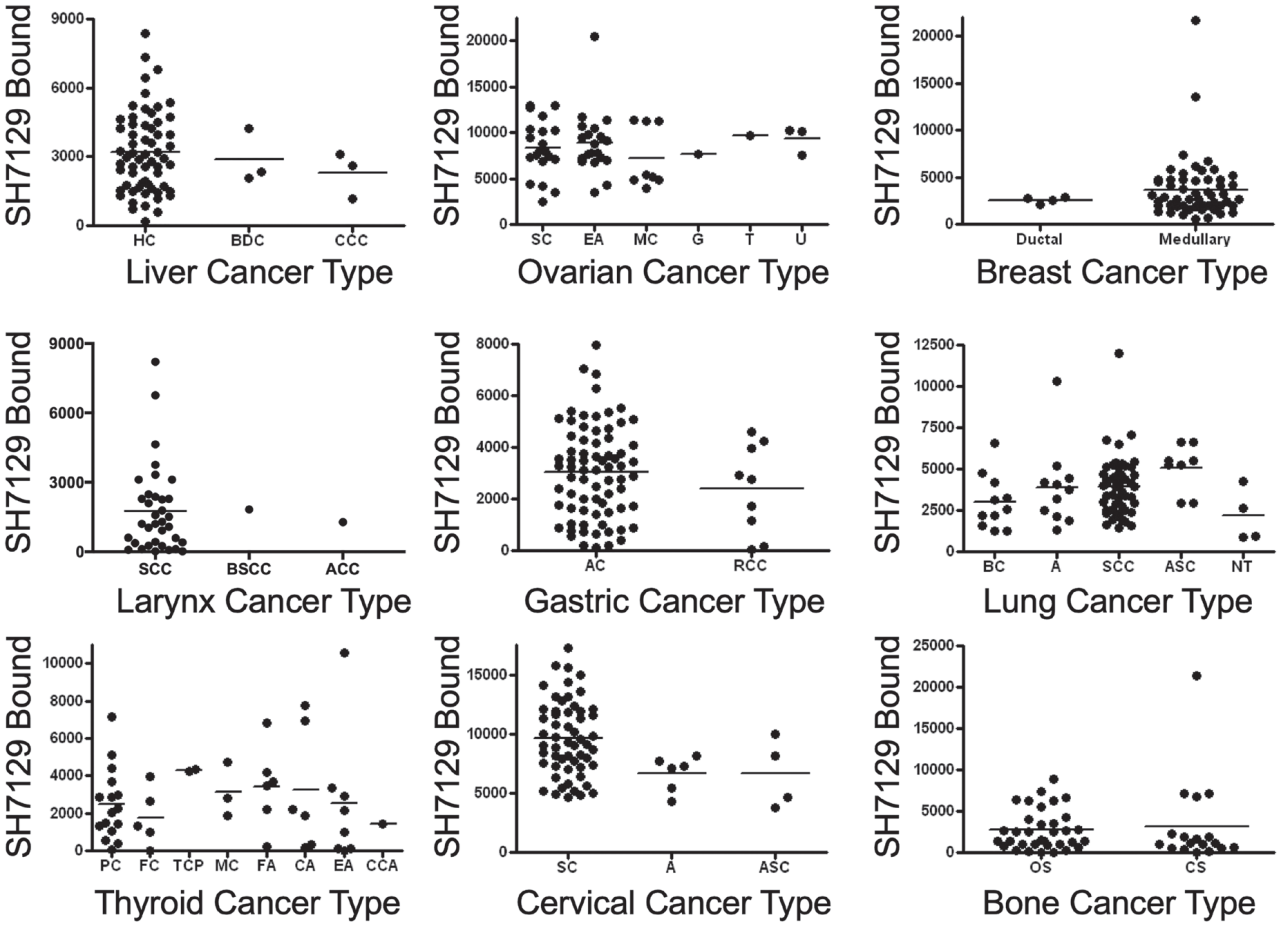
SH7129 binding to different types of nine non-lymphoid solid cancers. SH7129 binding data shown in Figure 8 were sorted by type for nine of the cancers and the binding to the different tumors within each type were plotted for comparison. SH7129 binding to the different types of lung, liver, ovarian, laryngeal, gastric, breast, and bone cancers were not found to be significantly different. In a number of cases there were two few cases to provide a meaningful comparison. A statistically significant difference was only observed for two types of cervical cancer; the squamous cell carcinomas (SC) bound more SH7129 than the adenocarcinoma (A) type (*p* = 0.006). Liver cancers: hepatocellular carcinoma (HC), bile duct carcinoma (BDC), and clear cell carcinoma (CCC). Ovarian cancers: serous cystadenocarcinoma (SC), endometrioid (EA), mucinous cystadenocarcinoma (MC), granulosa cell tumor (G), thecoma (T), and undifferentiated adenocarcinoma (U). Breast cancers: ductal and medullary. Larynx cancer: squamous cell carcinoma (SCC), basaloid squamous cell carcinoma (BSCC), and acinic cell carcinoma (ACC). Gastric cancers: adenocarcinoma (AC) and ring cell carcinoma (RCC). Lung cancers: bronchioloalveolar carcinoma (BC), adenocarcinoma (A), squamous cell carcinoma (SCC), adenosquamous carcinoma (ASC), and neuroendocrine tumor (NT). Thyroid cancers: papillary carcinomas (PC), follicular papillary carcinoma (FC), tall cell papillary carcinoma (TCP), medullary carcinoma (MC), follicular adenoma (FA), colloid adenoma (CA), embryonic adenoma (EA) and clear cell adenoma (CCA). Cervical cancers: squamous cell carcinoma (SC), adenocarcinoma (A), and adenosquamous carcinoma (ASC). Bone cancers: osteosarcoma (OS) and chondrosarcoma (CS).

SH7129 binding to the different types of nine of the non-lymphoid solid cancers analyzed (Figure 12) was also compared to determine if a particular type might express more or less of the HLA-DRs. With one exception, the level of target HLA-DR expression by the different types of lung, liver, ovarian, laryngeal, gastric, breast, and bone cancers were not found to be statistically different. Within the cervical cancers analyzed, the squamous cell carcinomas (SC) bound more SH7129 than the adenocarcinomas (A) (*p* = 0.006).

**Figure 13:**
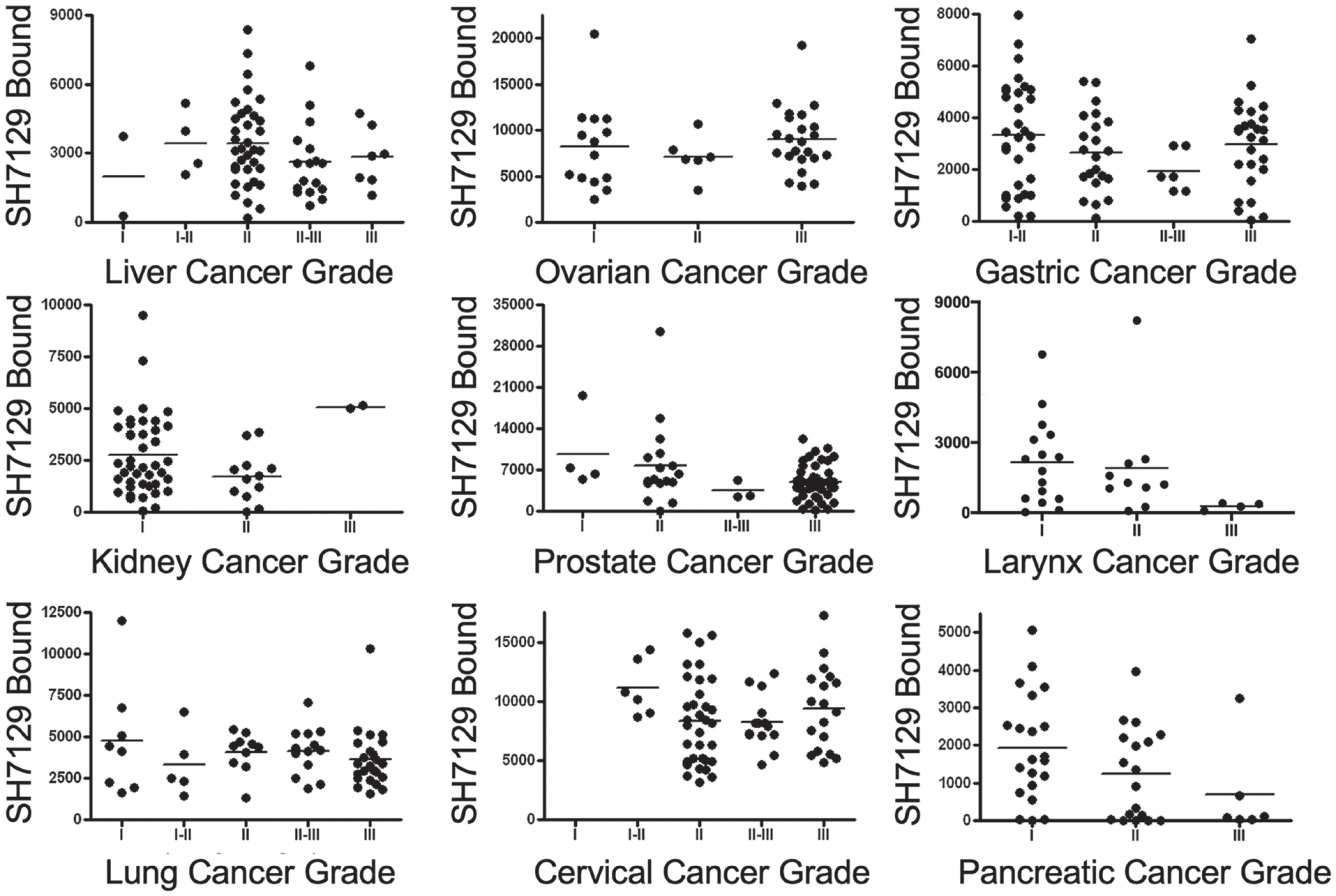
Comparison of SH7129 binding to nine cancers by grade. SH7129 binding data shown in Figure 8 were sorted by grade for nine of the cancers for which there was grade information, and the binding to the different tumors within each type were plotted for comparison. Statistical analyses of the data indicate there is no correlation between the amount of SH7129 bound and tumor grade in liver, ovarian, gastric, prostate, laryngeal, lung, cervical, or pancreatic cancers. The comparison suggested what appears to be a significantly higher level of SH7129 binding to grade III compared to grade II kidney cancers (*p* = 0.0350), but this result is based on the analysis of only two grade III cases.

A comparison of the amount of SH7129 bound to tumors classified by grade yielded a similar result (Figure 13). No difference in SH7129 binding was observed as a function of tumor grade in liver, ovarian, gastric, prostate, laryngeal, lung, cervical or pancreatic cancers. The comparison suggested what appears to be a significantly higher level of SH7129 binding to grade 3 compared to grade 2 kidney cancers (*p* = 0.0350), but this result is based on the analysis of only two grade 3 cases. While this difference may prove to be real, such a conclusion cannot be confirmed until a much larger number of cases are examined. It is important to point out that the same is true for many of the cancer types and grades analyzed. The results obtained from the comparisons involving only a few cases per cancer type or grade may not reflect the true variation that is present in the larger population.

## DISCUSSION

The SH7129 used to stain the tumor biopsy tissue sections in this study is a biotinylated analog of SH7139 that was synthesized for use in cell binding studies and as a potential companion diagnostic for prescreening patients to identify those with tumors that express the HLA-DRs SH7139 targets. The HLA-DR targeting domains of SH7139 and SH7129 are identical and the two molecules differ only in the effector (a DOTA chelating group or a biotin, respectively) conjugated to the free amine at the non-functional end of the scaffold that links the recognition elements together. The replacement of the DOTA by biotin has been reported in earlier studies to have no impact on either the selectivity of the targeting of HLA-DR [51] or its biological activity [51–53].

With the exception of a single section of lung, esophagus, prostate, stomach and heart tissue derived from a single tissue donor, SH7129 binding to normal tissues was very weak or not observed. Expression of the MHC class II protein HLA-DR is typically restricted to antigen presenting cells in a healthy individual [37, 176]. These include the B-lymphocytes, monocytes, macrophages and dendritic cells found in blood, tonsils, spleen, lymph nodes, thymus, and bone marrow, Kupffer cells in liver, microglia in brain, Langerhans cells in skin, other lymphocytes and macrophages that are frequently found infiltrating numerous organs (e.g., the kidney, breast, and lung), as well as certain epithelial or endothelial cells that provide the first line of an organ’s defense against infection or injury. HLA-DRs are expressed at relatively low levels in resting lymphocytes (approximately 1–2 × 10^5^ molecules per cell) [172].

Expression of HLA-DR in normal tissues that lack APCs does not typically occur except following injury or the onset of disease. Low levels of HLA-DR expression have been observed in cases of primary biliary cirrhosis [177], gastritis [139, 140], cutaneous graft-*vs*-host disease [178], prostatitis [179], and hyperthyroidism [142]. HLA-DR expression has also been associated with inflammations involving the lung [180], bowel [181, 182], pancreas [183], myocardial tissue [184], and skin [178] and with B-cell derived lymphomas, certain T-cell lymphomas, leukemias, myelomas, and a number of other solid cancers. Although HLA-DR is not normally expressed in skin [178, 185], it has been observed in keratinocytes in skin biopsies taken from individuals with psoriasis [186] and many other diseases caused by an overactive immune response that triggers inflammation, including lupus erythematosus, vitiligo, lymphocytic vasculitis, lichen sclerosus, morphea, lichen planus, erythema nodosum, granulomatous dermatoses, allergic dermatitis, various infectious dermatoses, and Sweet’s syndrome [187, 188].

As expected, normal lymphoid tissues containing APCs bound SH7129. The extremely low levels of SH7129 binding to zona reticularis (adrenal) and white matter (cerebellum) appear related to HLA-DR expression by adrenal cells destined for turnover [131] and microglia or fibrous astrocytes that take on the role of antigen presenting cells in the white matter of the cerebellum of the elderly [132–138] or following brain injury or the onset of neurological disease [132–134, 136, 189, 190]. In each of the isolated cases of a single normal tissue section showing HLA-DR expression, as well as the observed staining of hepatocytes and cells in the white matter of the cerebellum, the level of SH7129 binding to the expressed HLA-DR was only a small fraction of that observed for resting B-cell lymphocytes [54] and was so low that it could only be detected in tissue sections that were not counterstained. The staining of hepatocytes, which do not express HLA-DRs, may be explained by SH7129’s binding to the abundant OATP1B1 and OATP1B3 transporters present in liver.

Based on the results of a series of toxicology and safety studies conducted with SH7139 in dogs and rats at doses of the drug up to 4,000 and 12,000 times, respectively, of the anticipated therapeutic dose (data not shown), the low-level of SHAL binding to these normal tissues does not appear to have an adverse impact on their function. No organ or tissue in the treated animals showed any macroscopic or microscopic indication of pathology or toxicity, B-cell lymphocytes expressing low levels of HLA-DR were not adversely affected, the serum chemistry of the treated animals showed no evidence of liver or renal damage, there were no observed abnormalities in electrocardiography parameters (heart rate, RR interval, PR interval, QRS duration, QT interval or QTc interval), and the Functional Observational Battery performed to assess the central nervous system for pharmacological effects showed no indication of an adverse association with SH7139 exposure.

Consistent with previous reports of HLA-DR expression by a number of different lymphomas and leukemias [30, 37, 191–193], a significant fraction of each of the seven NHL subtypes tested in this study were found to express HLA-DRs that bind SH7129. In addition to providing additional confirmation that HLA-DR is expressed by most, if not all, B-cell lymphoma subtypes, the current results also show the majority of the tumors examined (56% of all cases tested) expressed HLA-DRs recognized by SH7129. Binding was observed in each of the anaplastic large cell lymphoma cases tested. Only about a quarter of the mantle cell lymphomas and one third of the follicular lymphomas were found to bind SH7129. Although the median level of binding was lower than that observed for the other NHL subtypes, one mantle cell lymphoma bound more SH7129 than any other NHL subtype.

Similar results were obtained for sixteen of the eighteen other solid cancers examined. Cervical, ovarian, prostate and colorectal cancers bound considerably more SH7129 than any of the lymphomas and many of the other solid cancers. SH7129 binding comparable to the levels found in NHL were observed in melanoma, kidney, breast, bladder, pancreatic, lung, bone, prostate, larynx, liver, gastric, and thyroid cancers. SH7129 binding was rarely detected in esophageal and head and neck cancers. As observed for the different types of NHL, a wide range in tumor to tumor variability in SH7129 binding was also observed for the majority of the non-hematological cancers expressing HLA-DR.

Although there were too few cases of the non-hematological cancer types represented in the tumor biopsy sets analyzed to be able to draw definitive conclusions regarding cancer type differences in SH7129 binding, the available data suggest differences in HLA-DR target expression and SH7129 binding amongst the different types of liver, larynx, thyroid, ovarian, gastric, cervical, lung, or bone cancers are likely to be small if they differ at all. Only the medullary carcinomas (MC) and invasive ductal carcinomas (IDC) of breast cancer were observed to differ in their expression of HLA-DRs recognized by SH7129. While the amount of SH7129 binding appears to be similar, based on the analysis of a very limited number of invasive ductal cases, the frequency with which the two types of breast cancer expressed the HLA-DR target was quite different. Approximately 4% of the IDC tumors were found to express the target HLA-DRs and bind detectable levels of SH7129. Two thirds of the MC tumors, in contrast, bound SH7129. The larger percentage of medullary carcinomas binding SH7129 in this study are consistent with reports by others who also show a much higher incidence of HLA-DR expression by medullary breast cancers, which typically have heavier and more frequent lymphocytic infiltration [81, 82], compared to invasive ductal carcinomas [194–197].

While SH7139 was originally developed as a therapeutic and imaging agent for B-cell derived lymphomas, the current results suggest SH7139 should also be considered as a viable alternative for treating many other types of cancer. Since the HLA-DR target is unique, SHALs can be used in combination therapies with most other drugs. Because cervical, ovarian and colorectal cancer express much higher levels of the HLA-DRs that bind SH7129, these cancers would be expected to respond even more favorably to SH7139 therapy than NHL. The cell surface HLA-DR molecules to which SH7139 binds are rapidly internalized [198], and tumor cells with significantly larger numbers of HLA-DR molecules on its surface would be expected to more rapidly accumulate SH7139 and achieve much higher intracellular concentrations of the drug.

One unexpected finding in our analysis of SH7129 binding to the non-hematological cancers was that a significant number of pancreatic and liver cancer cases bind the diagnostic, indicating they also express HLA-DRs targeted by SH7139. Although the median level of SH7129 binding was low compared to other cancers, it was similar to some types of NHL, such as the mantle cell lymphomas. Patients diagnosed with pancreatic, liver, and lung cancers currently have the poorest prognoses, with five-year survival rates of only 9%, 19%, and 20% respectively [199]. Therapies targeting the HLA-DRs expressed by pancreatic, liver and lung cancers, either alone or in combination with other drugs, could markedly improve the outcomes for patients diagnosed with these malignancies.

HLA-DR expression by tumor cells, as in normal antigen presenting cells, is often accompanied by the presentation of peptide antigens to T-cell lymphocytes [19, 67, 73, 200–202]. Among these antigens are peptides derived from proteins that bear mutations not found in normal cells [21, 22, 203–207], contain a unique conformational epitope or post-translational modification [208, 209], or whose expression is specific to the tumor cell [26, 210–212] or the normal cell from which the tumor developed [213–218]. One consequence of HLA-DR’s presentation of these abnormal or ‘non-self’ antigenic peptides to T-cells is the induction or enhancement of an immune response that targets the tumor producing the proteins from which the peptides originated [22, 200, 219]. Such responses have been reported to lead to systemic immunity [21] and better prognoses for patients with lymphoma [220], melanoma [221], colorectal [98, 100, 101, 222], gastric [111], ovarian [63] and certain types of laryngeal [107], and breast [81, 223, 224] cancers expressing HLA-DR than others diagnosed with same cancers that do not express HLA-DR. Tumors that do not express MHC class II proteins or have a deletion of the H2-DM gene which prevents the loading of antigenic peptides onto HLA-DRs containing CLIP [225] also appear to be better at escaping immunosurveillance [226–230] which has been suggested to lead to poorer tumor containment [228, 231–233], the outcome of which provides a worse prognosis for the patient [84, 100, 222, 224, 233–238]. While some exceptions have been reported [88, 239–241], this has led to the suggestion that one approach for improving cancer therapy might be to force all cancer cells to become antigen presenting cells [219, 226, 242]. Should such an approach be implemented as a means to trigger the patient’s immune system to mount a more effective antitumor response, it would also sensitize those tumors that do not normally express HLA-DR to SH7139 and other HLA-DR targeted therapeutics.

The correlation between HLA-DR expression and more positive outcomes for patients has not, however, been observed for all types of cancer. HLA-DR expression by acute myelogenous leukemias [241], myelomas [240] and diffuse large B-cell lymphomas [237] have been reported to be highly predictive of poor patient survival. These reports, combined with the fact that a large fraction of so many different types of cancer express HLA-DRs, suggest the presence of tumor associated HLA-DRs may in some cases confer a benefit to the growth or survival of cancer cells. At least two mechanisms that help cancer cells expressing HLA-DR avoid detection by the immune system have been described in support of this idea. Increased expression of HLA-DR by tumors in the absence of the co-stimulatory receptors CD80 or CD86, as one example, has been shown to suppress T-cell activation and tumor infiltration by lymphocytes. In the absence of these receptors the tumor not only evades detection, but its cells can more easily kill those lymphocytes that do invade tumor tissue [243]. Expression of the transcription regulator CD74, which always accompanies expression of HLA-DR, and its binding to the chemokine migration inhibitory factor (MIF) has also been shown to improve tumor cell survival by suppressing CD-44 mediated-apoptosis [244] and modulating other pathways that involve immune regulation and cell survival [245].

CD74, which is also called “Ii”, has a second function as a chaperone protein that is co-expressed along with HLA-DR to facilitate its proper folding, protect its antigen binding site from premature loading with peptides that will be presented to T-cells, and to direct the HLA-DR proteins to the late endosomal lysosomal antigen-processing compartments containing the antigenic proteins and peptides they will eventually present to T-cells [246]. In the endoplasmic reticulum, the α and β-subunits of HLA-DR bind to trimers of CD74 to form large nonameric complexes [247]. Following the transport of these complexes across the trans-Golgi network into late endosomal lysosomal antigen-processing compartments containing the antigenic proteins and peptides, the CD74 in the complex is processed by a series of proteolytic cleavages leaving the class II-associated Ii chain peptide (CLIP) bound inside the peptide binding site of HLA-DR. Upon removal of CLIP by HLA-DM, the antigenic peptides then bind inside the HLA-DR peptide binding site. In addition to those HLA-DR: CD74 complexes trafficked to the endosome, some of the complexes are also transported directly to the plasma membrane. Many of these complexes are under populated by HLA-DRs, leaving CD74 molecules on the surface of the cell that can bind to MIF and function as a transcription factor to promote tumor cell survival [245, 248].

The observation that HLA-DR expression by some cancers provides a benefit to the patient and its expression by other cancers provides a benefit to the tumor demonstrates the complexity of the cellular mechanisms that contribute to tumor cell survival and the immune system’s response to cells considered non-self. In the absence of other contributing factors, HLA-DR expression by tumors can stimulate an immune response targeting the tumor in those cases where the peptide antigens are immunogenic when presented to T-cells [21, 200, 219]. In cases where a sufficiently strong anti-tumor response cannot be induced by the antigens presented by HLA-DR, the co-expression of receptors such as CD74 that promote tumor cell survival may dominate the result. In other cases the outcome may be dictated by unrelated immunosurveillance evasion processes that come into play, such as increased production of T-reg lymphocytes [249], the secretion of immunosuppressive molecules (programmed death-ligand 1, TGF-β, indoleamine 2,3-dioxygenase, IL-10 or Fc receptor-like 6) that induce pathways that limit or repress the activity of cytotoxic T-cell lymphocytes or NK cells [250, 251], blocking of inhibitory checkpoints for immune activity [252], blockage of death receptor signaling [253, 254], downregulation or loss of MHC class I molecules [255–258], defects or alterations in MHC class I antigen processing [259] or interferon signal transduction, or the loss of gene function [260].

**Figure 14:**
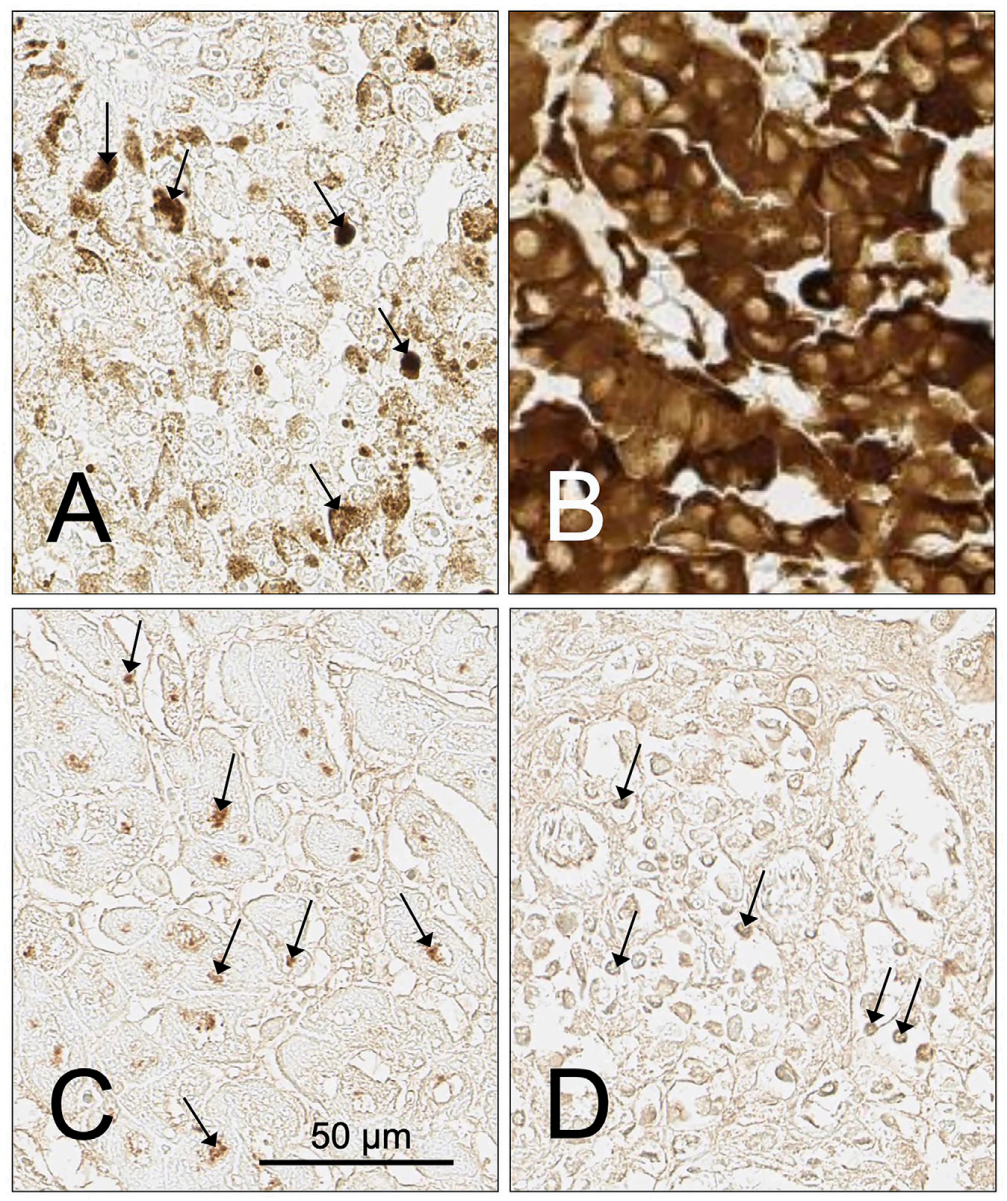
Examples of tumor cases with infiltrating lymphocytes or cells containing pigment that impact or prevent the assessment of SH7129 binding using DAB as a substrate. (**A**) Tissue sample ME481AB6, unstained moderately pigmented melanoma showing small pigment particles (arrows) similar in color to the insoluble product produced by SAHRP oxidation of DAB. (**B**) Tissue sample ME481AA8, unstained heavily pigmented melanoma section. (**C**) Tissue sample M672708, SLL/CLL tumor section showing SH7129 stained lymphocytes and macrophages (arrows). (**D**) Tissue sample BR807C3, breast medullary carcinoma section showing SH7129 stained infiltrating lymphocytes and macrophages (arrows). The images were captured at 40× magnification. The scale bar is the same for all images.

The SH7129 staining protocol used in this study provided a simple method to screen biopsy sections to identify tumors expressing the HLA-DRs SH7139 targets and to also obtain an estimate of the amount of SH7139 one might expect to bind to different tumors. In contrast to the Lym-1 antibody, which could also be used to detect the expression of a subset of HLA-DRs (including HLA-DR10), SH7129 works well for staining formalin fixed tissues. During our analysis of the samples, however, several limitations of the current method were identified that will need to be addressed as the diagnostic and staining protocol are developed further. One relates to the observation that the detection of SH7129 binding using horse-radish peroxidase and the current DAB substrate cannot be used to quantify HLA-DR target expression and SH7129 binding to tumor cells that are pigmented or contain significant amounts of melanin. The image analysis approach we used to quantify SH7129 binding to the tumor cells could not distinguish between the brown insoluble product formed by horse-radish peroxidase oxidation of the DAB substrate and the similarly colored melanin pigment present in a number of the melanoma biopsies (Figure 14A and 14B) or the melanin present in the basal keratinocytes found in normal skin tissue. Melanin does not fluoresce under visible or ultraviolet light excitation. One solution might be to replace SH7129 with a fluorochrome-tagged analog of SH7139, provided the presence of the fluorescent dye does not influence HLA-DR binding or alter the reagent’s selectivity.

Other factors, such as the presence of large numbers of tumor infiltrating lymphocytes (TILs), macrophages or dendritic cells that also express HLA-DR (Figure 14C and 14D), could make it difficult to accurately quantify SH7129 binding by the tumor cells. If the numbers of TILs in the section are small, the impact will be negligible. But if large numbers of these cells are present in the tissue, the extent of SH7129 binding to the tumor cells can only be determined in areas free of the antigen presenting cells. The lack of structural uniformity within the tissue being imaged can also present quantification issues. While all cores in a tumor microarray are usually cut to the same thickness, differences in thickness can contribute to variation in the amount of cellular material that is stained and the amount of diagnostic bound. This is particularly important when the target protein, such as HLA-DR, is not only found bound to the membrane but is also abundant in the cytoplasm. Local variation in the optical properties of unstained cells and supporting or connective tissue also contribute background absorption and attenuation of the light transmitted through the tissue that adds to the signal provided by the oxidized product of the DAB substrate. Tumor to tumor differences in density of cells packed within the section, disruptions in the integrity of the core (tears or missing tissue) or the presence of vacuoles, capillaries, or necrotic regions can change the amount of tumor tissue that is available to bind the diagnostic. The use of sequentially cut cores for the stained and unstained control slides should help minimize these contributions to variability and reduce errors in the quantification of SH7129 binding to tumor cells.

## MATERIALS AND METHODS

### Chemicals and reagents

The chemicals and reagents used in this study were obtained from the following sources: Key Organics, Camelford, UK (4-[4-[(4-chlorophenyl) methyl] piperazin-1-yl]-3-nitrobenzoic acid and 3-[2-[3-chloro-5-(trifluoromethyl) pyridin-2-yl] oxyanilino]-3-oxopropanoic acid), Sigma-Aldrich, St. Louis, MO (3,3′-diaminobenzidine tetrahydrochloride, hydroxybenzotriazole, trifluoroacetic acid, anhydrous dimethylformamide, 2-(1*H*-benzotriazol-1-yl)-1,1,3,3-tetramethyluronium hexafluorophosphate, Wang trityl chloride resin, phosphate buffered saline, citrate buffer, hematoxylin, xylene, ethanol), Macrocyclics, Plano, TX, USA (DOTA NHS ester), TCI, Shanghai, China (Dabsyl chloride), GL Biochem, Shanghai, China (Fmoc-D-Lys (Boc)-OH, Fmoc-D-Lys (Dde)-OH), Fmoc-L-Val-OH), Biomatrik, Zhejiang, China (Fmoc-8-amino-3,6-dioxaoctanoic acid), Electron Microscopy Sciences, Hatfield, PA, USA (Permount, 30% hydrogen peroxide), VWR Scientific, Radnor, PA, USA (acetonitrile, biotin N-hydroxysuccinimide ester, N, N-diisopropylethylamine) Roche Molecular Diagnostics, Pleasanton, CA, USA (Ventana Endogenous Biotin Blocking kit), Leica Biosystems Inc, Buffalo Grove, IL, USA (Leica BOND RX Automated Slide Stainer reagents) and Vector Labs, Burlingame, CA, USA (streptavidin conjugated horse-radish peroxidase).

### Synthesis of the biotin analog of SH7139

SH7129 was synthesized and purified by AmbioPharm Inc., (North Augusta, SC, USA) using a modification of the procedure described previously [51, 52]. Briefly, the SHAL was synthesized using solid phase chemistry by the stepwise attachment to a Wang resin of Fmoc-D-Lys#1(Boc)-OH, Fmoc-AEEA-OH#1 (Fmoc-8-amino-3,6-dioxaoctanoic acid), Fmoc-D-Lys#2(Dde)-OH, Fmoc-AEEA-OH#2, Fmoc-L-Val-OH, and Dabsyl chloride using standard Fmoc (N-9-fluorenylmethoxycarbonyl) chemistry with HBTU (2-(1*H*-benzotriazol-1-yl)-1,1,3,3-tetramethyluronium hexafluorophosphate)/HOBt (Hydroxybenzotriazole)/DIPEA (N, N-Diisopropylethylamine) as the coupling reagents. The side chain amino group of D-Lys#2-(Dde)-OH was deprotected with 4% hydrazine in dimethylformamide (DMF) and then coupled to Fmoc-D-Lys#3-(Dde)-OH using the same coupling procedure. Fmoc-AEEA-OH#3 was next coupled to deprotected D-Lys#3(Dde) and 4-(4-(4-chlorobenzyl) piperazine)-3-nitrobenzenecarboxylic acid (Cb ligand) was then linked to the deprotected AEEA-OH#3 using the same coupling procedure. The third ligand Ct (3-(2-((3-chloro-5-(trifluoromethyl)-2-pyridinyl) oxy)-anilino)-3-oxopropanoic acid) was then attached to the deprotected ε-amine of D-Lys#3. The assembled free amine form of the SHAL was cleaved from the resin, deprotected and subsequently precipitated as a crude solid. The crude product was purified by standard RP-HPLC methods and isolated by lyophilization. Biotin was attached to the free amine on the terminal lysine by dissolving the SHAL in anhydrous DMF, N, N-Diisopropylethylamine (DIEA) and adding solid biotin N-hydroxysuccinimide ester (biotinyl-OSu). The mixture was nutated for 15 min, and the reaction was monitored by analytical HPLC. Upon completion, the reaction solution was diluted with a small volume of water/acetonitrile (50/50) containing 1% trifluoroacetic acid (TFA) and purified by HPLC. The purified SH7129 was lyophilized and then analyzed by LC/MS and NMR spectroscopy to determine its purity (96.2%) and confirm its molecular mass (2165.6 Da) and structure, respectively.

### Tissue and tumor microarrays

The normal human tissue microarrays (FDA808-1 and FDA808-2) containing fixed and paraffin embedded sections of twenty-four different tissues obtained from three individuals and tumor microarrays (TMAs) containing fixed and paraffin embedded tumor biopsy sections obtained from patients diagnosed with different non-Hodgkin’s lymphoma subtypes (LM241, LM242, LM482, LY804, LY1001b, NHL801, NHL802, NHL803, OD-CT-LyMly02) and other solid cancers (BC07014a, BC08118, BC041115d, BC12011b, BM483, BR807, CXC1501, ESC1021, HN483, LP801, LUC1021, LVC1501, Me481a, Me1004e, OD-CT-DgCol04, OD-CT-DgStm01, OD-CT-LyMly02, OS802, OVC1501, PA961c, PR803c, T111, T291, TH802) were obtained from U. S. Biomax (Rockville, MD, USA). An additional set of diffuse large B-cell lymphoma, mantle cell lymphoma, follicular lymphoma and SLL/CLL TMAs were obtained from Dr. John G. Gribben, Barts Cancer Institute, Queen Mary University of London, Charterhouse Square, London, UK.

### SH7129 staining protocol

SH7129 was prepared as a stock solution by dissolving 10 mg of the dry compound in 1 ml dimethyl sulfoxide. The formalin fixed normal tissue and tumor microarrays were stained using a Leica BOND RX Automated Slide Stainer (Leica Biosystems Inc., Buffalo Grove, IL, USA) to maximize slide-to-slide uniformity in staining and processing. The fixed slides were deparaffinized using the Leica dewax solution, rehydrated with an alcohol series (100%, 95%, 70% and 30% for 4 min each) followed by antigen retrieval in citrate buffer at pH 6 and 90°C for 20 min. After performing a 5 min hydrogen peroxide block, the slides were washed three times with BOND Wash Solution, endogenous biotin was blocked using the Ventana Endogenous Biotin Blocking kit (Roche Molecular Diagnostics, Pleasanton, CA, USA), the slides were washed three additional times with BOND Wash Solution, and then stained with SH7129 (100 μg/ml in PBS, 1% DMSO) for 30 min. Following three washes with BOND Washing Solution, the slides were treated with Streptavidin-horse radish peroxidase (SAHRP) for 30 min, washed 3 times with BOND Wash Solution and once with deionized water, treated with Mixed DAB (3,3-diaminobenzidine) Refine for 10 min, and then washed four times with deionized water, once with BOND Wash Solution and a final deionized water wash as per the BOND Polymer Refine IHC protocol (Histowiz Inc., Brooklyn, NY, USA). The SH7129 stained tumor microarray slides were not counterstained with hematoxylin. The slides were then dehydrated by immersion in an alcohol series (30%, 70%, 95% and 100% for 4 min each), cleared with xylene and mounted with Permount.

### Analysis of SH7129 binding to NHL and other solid tumors

Whole slide images of the SH7129 stained and control (duplicate slide cut from same core treated with PBS instead of SH7129) normal tissue and tumor microarrays were captured at 40× magnification using an Aperio AT2 Digital Pathology Scanner (Leica Biosystems, Buffalo Grove, IL, USA). SH7129 binding or lack of binding to the cells was confirmed by visual inspection. Cells expressing the HLA-DRs that bind SH7129 showed stain associated with both the membranes and cytoplasm. Cell-to-cell variation in SH7129 binding was determined by performing densitometric analyses of fifty (50) individual tumor cells from a representative moderate to high SH7129 binding tumor for six of the NHL subtypes using NIH ImageJ version 1.42 software [262]. An effort was made during the analysis to include cells representing the full range of SH7129 binding. Individual cell data were not obtained for the small lymphocytic lymphomas due to our inability to accurately define the individual cell boundaries in these tumors. To estimate the tumor-to-tumor variation in SH7129 binding, additional lower magnification digital images containing the array of cores for the two slides (SH7129 stained and control without SH7129) were captured at 10× magnification, the images were inverted, and the amount of bound SH7129 was determined by densitometric analysis of each tumor section using the same NIH ImageJ software. Integrated density data were collected from a 384-pixel area of each core and from ten blank (background) 384-pixel areas distributed across the slide near or between the cores. Core sections containing voids or tears (missing tissue), lacking a corresponding core in the control slide, or obtained from pigmented tumors were not analyzed. In cases where there were duplicate or triplicate cores for each biopsy on the slides, the data obtained from the analyses of the replicates were averaged. The amount of bound SH7129 (per 384-pixel area) was then calculated for each biopsy sample as follows:

Bound SH7129 = (IntDen_SH7129_ – IntDen_SH7129Bkg_) – (IntDen_NoSH7129_ – IntDen_NoSH7129Bkg_)

where IntDen_SH7129_ is the integrated density of the biopsy section stained with SH7129, IntDen_SH7129Bkg_ is the mean of the integrated densities of the ten blank regions of the SH7129 stained slide, IntDen_NoSH7129_ is the integrated density of the biopsy section that was processed for staining without SH7129, and IntDen_NoSH7129Bkg_ is the mean of the integrated density of the ten blank regions of the control slide processed for staining without SH7129.

The data were analyzed and plotted using GraphPad Prism version 8.1.2. Statistical analyses of two groups of data were performed using an unpaired *t-test*. Analyses of three or more groups of data in which the standard deviations of the groups were similar were analyzed using a one-way ANOVA test followed by Tukey’s multiple comparison test. Data sets containing three or more groups in which the standard deviations of the groups being compared were different were analyzed using both a Brown-Forsythe and Welch’s ANOVA test followed by Dunnett’s T3 multiple comparisons test. In both cases in which three or more groups were compared, the assumption was made that the data fit a Gaussian distribution based on the observation that the amount of SH7129 bound per biopsy case did roughly fit a Gaussian distribution.

## CONCLUSIONS

The tumor biopsy binding studies conducted with SH7129 have shown the HLA-DRs targeted by SH7139 are expressed by many different types of cancer. A significant fraction of the cases in each of the seven subtypes of B-cell lymphomas were found to express these HLA-DRs, as indicated by the binding of SH7129, and the range of expression varied by as much as 10 to 100-fold. Plasma cell myelomas and seventeen other types of non-hematological cancers have also been found to express the HLA-DRs recognized by these SHALs, with some at levels much higher than many types of NHL. Cervical, ovarian, prostate and colorectal cancers bound the most SH7129, followed by non-Hodgkin’s lymphomas, plasma cell myelomas, lung, liver, bone, kidney, thyroid, melanoma, breast, laryngeal, gastric, pancreatic and bladder cancers. Only a few cases of head and neck and esophageal cancers bound SH7129. Comparisons of SH7129 binding to different types of nine non-hematological cancers only revealed a significant difference for two types of cervical and two types of breast cancer. SH7129 binding did not correlate well with tumor grade in the nine cancers for which data was available. The results obtained in this study also provide additional evidence of SH7129’s utility as a diagnostic to identify tumors expressing the HLA-DRs targeted by SH7139 and to estimate the magnitude of their expression. SH7129 should prove useful in future clinical trials as a surrogate biomarker detection reagent for screening formalin-fixed paraffin embedded biopsy sections to identify patients with HLA-DR expressing tumors targeted by SH7139, Lym-1 antibody in Lym-1 CAR T-cell therapy clinical studies [261], and SHALs administered in combination with other drugs to individualize therapy and optimize treatment protocols for each patient. The observation of moderate to high levels of HLA-DRs expression by so many of the tumors tested suggest there are patients diagnosed with many cancers, in addition to NHL, that may benefit from the development of oncology drugs that target HLA-DR.

## Abbreviations

AEEA: 8-amino-3,6-dioxaoctanoic acid
ALCL: anaplastic large cell lymphoma
ANOVA: analysis of variance
BL: Burkitt lymphoma
Cb: 4-[4-[(4-chlorophenyl)methyl]piperazin-1-yl]-3-nitrobenzoic acid
CD: cluster of differentiation
Ct: 3-[2-[3-chloro-5-(trifluoromethyl)pyridin-2-yl]oxyanilino]-3-oxopropanoic acid
Da: Daltons
DAB: 3,3′-diaminobenzidine
Dde: N-(1-(4,4-dimethyl-2,6-dioxocyclohexylidene)ethyl)
DIEA: Diisopropylethylamine
DIPEA: N,N-Diisopropylethylamine
DLBCL: diffuse large B-cell lymphoma
DMF: dimethylformamide
DOTA: 1,4,7,10-tetraazacyclododecane-1,4,7,10-tetraacetic acid
Dv: 4-(Dimethylamino)azobenzene-4'-sulfonyl-L-valine
FL: follicular lymphoma
Fmoc: fluorenylmethoxycarbonyl
HAMA: human antibody-mouse antibody
HBTU: 2-(1*H*-benzotriazol-1-yl)-1,1,3,3-tetramethyluronium hexafluorophosphate
H&E: hematoxylin and eosin
HED: human equivalent dose
HLA-DR: human leukocyte antigen-DR type
HOBT: Hydroxybenzotriazole
HPLC: high performance liquid chromatography
IDC: invasive ductal carcinoma of the breast
IHC: immunohistochemistry
IND: investigational new drug
LC: liquid chromatography
LC/MS: liquid chromatography-mass spectrometry
MALT: mucosa-associated lymphoid tissue
MC: medullary carcinoma of the breast
MCL: mantle cell lymphoma
MHC: major histocompatibility complex
NHL: non-Hodgkin’s lymphoma
NMR: nuclear magnetic resonance
OATP: organic anion transporter polypeptide
OSu: N-hydroxysuccinimide ester
PBS: phosphate buffered saline
SAHRP: streptavidin horse-radish peroxidase
SHAL: Selective high affinity ligand
SLL: small lymphocytic lymphoma
TFA: trifluoroacetic acid
TILs: tumor infiltrating lymphocytes
TMAs: tumor microarrays
